# A comparative study of oral and gut microbiota for differentiating benign pulmonary nodules from lung cancer

**DOI:** 10.3389/fmicb.2026.1824419

**Published:** 2026-05-19

**Authors:** Zhe Zhao, Shupan Zhao, Fen Liu, Gang Li, Xing Hu, Lu Li

**Affiliations:** 1 Department of Medical Oncology, Beijing Hospital, National Center of Gerontology, Institute of Geriatric Medicine, Chinese Academy of Medical Sciences, Beijing, China; 2 Department of Health Service, Guard Bureau of the Joint Staff Department, Beijing, China

**Keywords:** 16S rRNA sequencing, biomarker, gut microbiota, gut-lung axis, lung cancer, oral microbiota, pulmonary nodules

## Abstract

**Objective:**

The differentiation between benign pulmonary nodules and early-stage lung cancer remains a significant clinical challenge due to the lack of highly specific non-invasive biomarkers. While the gut-lung axis has emerged as a critical player in pulmonary diseases, the concurrent evolution of oral and gut microbiota across the spectrum from benign nodules to lung cancer remains poorly characterized. This study aimed to investigate the compositional and dynamic changes in oral and gut microbiota across different stages of lung carcinogenesis, with particular emphasis on distinguishing tumor-specific alterations from nodule-related changes by incorporating benign nodules as a critical control group.

**Methods:**

In this retrospective case-control study, we enrolled 111 participants, including healthy controls (C, *n* = 36), individuals with benign pulmonary nodules (B, *n* = 18), treatment-naive early-stage lung cancer patients (EL, *n* = 20), and treatment-naive advanced-stage lung cancer patients (AL, *n* = 37). Saliva and fecal samples were collected for 16S rRNA gene sequencing (V3-V4 region). Microbial diversity, community composition, and differential taxa were analyzed using QIIME, LEfSe, and trend tests. Diagnostic efficacy of candidate markers was evaluated by ROC curve analysis.

**Results:**

Lung cancer was associated with significant remodeling of both oral and gut microbiota. While salivary α-diversity remained stable between groups, gut microbial richness (Chao1 index) was significantly increased in lung cancer patients compared to non-tumor individuals (*P* = 0.009). β-diversity analysis revealed distinct separation in community structure between non-tumor and lung cancer groups for both saliva and feces (*P* < 0.01). Trend analysis across the C→ B→ EL→AL continuum demonstrated significant gradient changes: salivary *Pasteurellaceae* (primarily *Haemophilus*) and Enterobacterales progressively decreased, while Actinobacteriota, *Rothia mucilaginosa*, and *Prevotella oris* significantly increased with disease progression. In the gut, *Veillonellaceae* and *Dialister* gradually declined, whereas *Bacteroides coprocola* exhibited a strong positive correlation with disease stage (ρ = 0.84, *P* < 0.001). The benign nodule group displayed an intermediate microbial phenotype. Notably, *R. mucilaginosa* and *Haemophilus* in saliva achieved AUCs of 0.851 and 0.884, respectively, in distinguishing non-tumor individuals from lung cancer patients, while gut *B. coprocola* yielded an AUC of 0.901, with maintained discriminatory performance in differentiating benign nodules from early-stage lung cancer.

**Conclusion:**

Lung cancer progression is accompanied by stage-specific remodeling of the oral-gut microecology. The incorporation of benign nodule controls enables the identification of tumor-specific microbial alterations with high diagnostic efficacy. Salivary *Rothia mucilaginosa* and gut *Bacteroides coprocola* represent promising non-invasive biomarkers for early lung cancer detection and benign-malignant discrimination, warranting further validation in multicenter prospective cohorts.

## 1 Introduction

Lung cancer remains one of the most prevalent and deadliest malignancies worldwide, posing a significant threat to human health. According to GLOBOCAN statistics, both the incidence and mortality rates of lung cancer rank highest among all cancer types (Bray et al., 2018; Torre et al., 2016). Despite continuous advancements in clinical diagnosis and treatment, the overall prognosis for lung cancer patients remains poor, largely attributable to low rates of early detection and a lack of effective screening modalities. Concurrently, the widespread adoption of imaging techniques such as low-dose computed tomography (LDCT) has led to a steady increase in the detection rate of pulmonary nodules (Torre et al., 2016), making their identification increasingly common even in routine health check-ups. This trend, however, presents a substantial clinical challenge: how can benign pulmonary nodules be reliably distinguished from early-stage lung cancer? The current absence of highly specific, non-invasive biomarkers means that many patients with benign nodules undergo unnecessary invasive procedures, while others with early-stage malignancies may miss the optimal window for intervention due to prolonged surveillance. Consequently, there is an urgent need to identify non-invasive biomarkers capable of facilitating early detection and enabling accurate benign-malignant discrimination.

In recent years, the role of the human microbiota in tumorigenesis and tumor progression has garnered considerable academic interest. The proposition of the “Gut-Lung Axis” theory has unveiled a bidirectional regulatory mechanism between the intestinal microbiota and pulmonary diseases (Dapito et al., 2012). Existing research indicates that gut dysbiosis not only affects digestive function but can also exert profound effects on extra-intestinal tumors, including lung cancer, through immune modulation and the translocation of metabolites. For instance, Dapito et al. demonstrated that the intestinal microbiota influences the progression of hepatocellular carcinoma via the TLR4 signaling pathway (Dapito et al., 2012); in the field of lung cancer, studies by Zheng et al. (2020) have confirmed significant differences in gut microbiota composition between lung cancer patients and healthy individuals, with specific microbial signatures even showing potential for predicting early-stage lung cancer. Beyond the gut microbiota, the oral cavity, serving as the common entry point for both the digestive and respiratory tracts, harbors microbial communities whose colonization status is intimately linked to pulmonary health. Oral microorganisms may enter the lower respiratory tract via micro-aspiration or the bloodstream, thereby potentially reshaping the pulmonary microenvironment.

However, most current research primarily focuses on a binary comparison between “confirmed lung cancer patients” and “healthy individuals,” often overlooking the critical intermediate clinical state of “benign pulmonary nodules.” Furthermore, few studies simultaneously investigate the co-evolutionary patterns of the oral microbiota (at the respiratory tract entry) and the gut microbiota (an immune regulation hub) across different stages of lung cancer progression (Shome et al., 2023; Sun et al., 2023; Yang et al., 2018; Yuan et al., 2023). This gap in study design makes it difficult to discern whether specific microbial alterations are cancer-specific drivers or merely epiphenomena accompanying non-specific pulmonary inflammation or the nodules themselves.

Against this background and driven by the identified clinical challenges, the present study was designed to address critical gaps in the literature. Unlike conventional investigations confined to healthy comparisons, we deliberately incorporated individuals with benign pulmonary nodules as an essential component of the non-tumor group, thereby enabling the differentiation of tumor-specific microbial alterations from non-specific inflammatory or nodule-related changes. Furthermore, by stratifying lung cancer patients into treatment-naive early-stage and advanced-stage subgroups, we sought to delineate the dynamic evolution of the microbial ecosystem across increasing tumor burden. Employing 16S rRNA gene sequencing of concurrently collected saliva and fecal samples, this study aimed to: (i) systematically compare the diversity and compositional profiles of oral and gut microbiota among healthy controls, benign nodule individuals, and patients at different lung cancer stages; (ii) identify specific bacterial taxa distinguishing non-tumor individuals from lung cancer patients using LEfSe analysis; and (iii) explore potential functional pathways through which microbial metabolites may contribute to lung cancer pathogenesis. The findings are expected to provide novel microbiological insights and identify potential biomarkers to facilitate non-invasive auxiliary diagnosis of lung cancer grounded in the gut-lung axis framework.

## 2 Materials and methods

### 2.1 Study design and population

**FIGURE 1:**
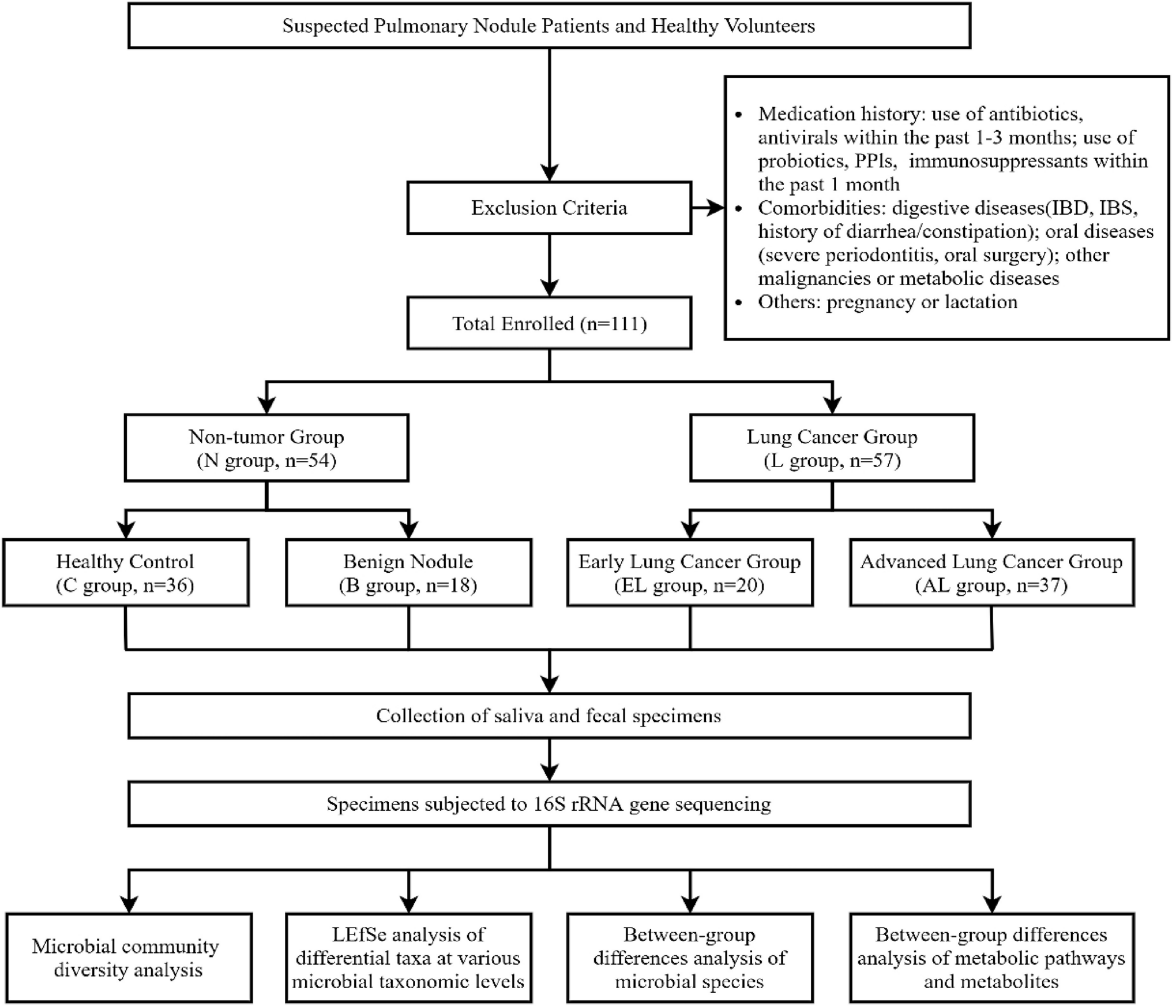
Flowchart of study participant enrollment and grouping strategy.

This study employed a retrospective case-control design. A rigorous enrollment and screening process was implemented to minimize confounding factors such as antibiotic usage and comorbidities, as depicted in Figure 1. A total of 111 participants who met the predefined inclusion and exclusion criteria were enrolled in Beijing between February 2023 and December 2023. Due to the retrospective nature of the study and the application of stringent exclusion criteria to control for confounding factors, the sample size was determined by the total number of eligible cases available during the study period. Although the sample size of the benign nodule group was relatively small (*n* = 18), post-hoc power analysis (G*Power v3.1.9) based on the observed large effect sizes (*f*^2^ > 0.35) for key discriminatory genera, such as gut B. coprocola, indicated that the study achieved a statistical power exceeding 80% (α = 0.05) to detect significant differences between the benign and lung cancer groups, confirming sufficient capacity for robust subgroup comparisons.

Based on clinical diagnosis and pathological findings, participants were categorized into four subgroups: healthy control (C, *n* = 36), benign pulmonary nodule (B, *n* = 18), treatment-naive early-stage lung cancer (EL, *n* = 20), and treatment-naive advanced-stage lung cancer (AL, *n* = 37). The benign nodule group was specifically included to distinguish cancer-specific microbiome alterations from non-malignant nodular changes. Early-stage lung cancer was strictly defined as AJCC 8th edition stage I-II disease, corresponding to T1-3, N0-1, M0. No patient with stage III or IV disease was included in this subgroup. To further clarify the clinicopathological distribution of the enrolled cohort, all patients in the early-stage group (*n* = 20) met the AJCC 8th edition criteria for stage I-II disease. Their TNM distribution was as follows: T1 (*n* = 11), T2 (*n* = 7), and T3 (*n* = 2); nodal status was N0 in 17 cases and N1 in 3 cases; no distant metastasis was identified in this group (M0 in all cases). In contrast, the advanced-stage group (*n* = 37) showed greater tumor burden, including T1-2 (*n* = 9), T3 (*n* = 14), and T4 (*n* = 14) disease; nodal involvement was distributed as N0-1 in 9 patients and N2-3 in 28 patients. Regarding metastatic status, 15 patients had no distant metastasis (M0), whereas 22 had distant metastasis (M1), involving common sites such as bone, brain, adrenal gland, and liver. Importantly, no patient in this cohort had intestinal metastasis or other gastrointestinal tract involvement. Patients with known gastrointestinal metastasis were excluded a priori to minimize the possibility that the observed gut microbial alterations were driven by direct intestinal tumor colonization rather than systemic tumor progression.

### 2.2 Inclusion and exclusion criteria

#### 2.2.1 Inclusion criteria

(1) Lung Cancer Group: Patients were diagnosed with primary lung cancer based on histopathological or cytological examination; had not received any anti-tumor therapy (including surgery, chemotherapy, radiotherapy, targeted therapy, or immunotherapy) prior to sample collection; had complete clinical TNM staging data, encompassing patients from stage I to IV (corresponding to the “treatment-naive early-stage” and “treatment-naive advanced-stage” subgroups in this study); were aged ≥ 18 years with intact consciousness.
(2) Benign Pulmonary Nodule Group: Patients presented with pulmonary nodules on chest CT; nodules were confirmed as benign lesions (e.g., hamartoma, inflammatory pseudotumor) by histopathology, or showed no significant change over a minimum of 2 years of clinical follow-up, with malignancy clinically ruled out; had no history of other malignancies.
(3) Healthy Control Group: Participants were recruited from concurrent health check-up populations, presenting with essentially normal findings on physical examination and routine laboratory tests; chest X-ray or CT revealed no pulmonary nodules, inflammation, or other organic lesions.

#### 2.2.2 Exclusion criteria

(1) Pharmacological Interference: Use of microecological modulators such as probiotics, prebiotics, or yogurt products within 1 month prior to sample collection; long-term use of proton pump inhibitors (PPIs), immunosuppressants, or corticosteroids.
(2) Comorbidity Interference: History of inflammatory bowel disease (IBD, e.g., Crohn’s disease, ulcerative colitis), irritable bowel syndrome (IBS), recent severe diarrhea or constipation, or gastrointestinal surgery; diagnosis of severe periodontitis, gingivitis, oral mucosal lesions, or recent dental surgery; presence of other malignancies, autoimmune diseases (e.g., systemic lupus erythematosus, rheumatoid arthritis), severe metabolic diseases (e.g., poorly controlled diabetes), or severe cardiac, hepatic, or renal insufficiency.
(3) Pregnancy or lactation.

### 2.3 Grouping

The 111 participants were divided into the non-tumor group (Group N, *n* = 54) and the lung cancer patient group (Group L, *n* = 57). Group N consisted of individuals with no history of malignancy and was further subdivided into two subgroups: the healthy control subgroup (Control, *n* = 36): healthy volunteers with normal physical examination indicators and no pulmonary nodules or other significant organic lesions; and the benign pulmonary nodule subgroup (Benign, *n* = 18): patients diagnosed with benign pulmonary nodules via imaging or pathology, included to control for non-specific microbial alterations caused by the nodules themselves.

Group L was divided according to the AJCC 8th edition TNM staging system into: the treatment-naive early-stage lung cancer subgroup (*n* = 20), including only patients with stage I-II disease; and the treatment-naive advanced-stage lung cancer subgroup (*n* = 37), including patients with stage III-IV disease. No patient with intestinal metastasis was included in either subgroup.

### 2.4 Sample collection and processing

#### 2.4.1 Specimen collection and sequencing strategy

All participants provided samples following standardized procedures. Each participant provided 1–2 saliva and/or fecal specimens. A total of 283 valid biological samples were ultimately collected, comprising 147 saliva samples and 136 fecal samples. Genomic DNA was uniformly extracted from the collected microbial samples. Specific primers targeting the V3-V4 hypervariable region of the bacterial 16S rRNA gene were used for PCR amplification to construct sequencing libraries. Subsequently, high-throughput sequencing was performed to obtain data on microbial community composition and diversity.

#### 2.4.2 Genomic DNA extraction and PCR amplification

Saliva and fecal samples were immediately stored at −80°C after collection. Total genomic DNA was extracted using the CTAB/SDS method. DNA concentration and purity were assessed by 1% agarose gel electrophoresis and Qubit 2.0 fluorometric quantification. Only DNA samples meeting the following quality criteria were used for downstream analysis: intact high-molecular-weight bands without obvious degradation on agarose gel, A260/A280 ratio of 1.8–2.0, and DNA concentration ≥ 10 ng/μL. Qualified DNA was normalized to a working concentration of 10 ng/μL using sterile nuclease-free water, and approximately 10 ng of template DNA was used for each PCR reaction. PCR amplification targeted the V3-V4 hypervariable region of the bacterial 16S rRNA gene. Specific primers with barcode sequences were used, as follows:

Forward primer (341F): 5’-CCTAYGGGRBGCASCAG-3’;

Reverse primer (806R): 5’-GGACTACNNGGGTATCTAAT-3’.

The PCR reaction mixture (30 μL) comprised: 15 μL Phusion^®^ High-Fidelity PCR Master Mix (New England Biolabs), 0.2 μM of each primer, and approximately 10 ng of template DNA. The thermal cycling protocol was: initial denaturation at 98°C for 1 min; 30 cycles of denaturation at 98°C for 10 s, annealing at 50°C for 30 s, and extension at 72°C for 30 s; and a final extension at 72°C for 5 min.

PCR products were examined by 2% agarose gel electrophoresis, and the main bands were purified using the GeneJET Gel Extraction Kit (Thermo Scientific). Sequencing libraries were constructed using the NEB Next^®^ Ultra™ DNA Library Prep Kit for Illumina (NEB, United States). Library quality was assessed with the Qubit 2.0 Fluorometer (Thermo Scientific) and the Agilent Bioanalyzer 2100 system; only libraries with a single expected peak and no adapter contamination or primer-dimer signal were subjected to sequencing. Finally, libraries were sequenced by Novogene Co., Ltd. (Beijing, China) on the Illumina NovaSeq platform, generating 250 bp paired-end reads.

#### 2.4.3 Bioinformatics analysis

Paired-end reads were assigned to samples based on their unique barcodes and primer sequences and merged using FLASH (V1.2.7) to obtain raw tags. Quality filtering was performed using the QIIME (V1.9.1) pipeline to remove low-quality sequences, yielding high-quality clean tags. Sequence clustering into operational taxonomic units (OTUs) was performed using Uparse software (v7.0.1001) with a default 97% identity threshold. Representative sequences for each OTU were selected and annotated for taxonomic information (kingdom, phylum, class, order, family, genus, species) using the Mothur method against the SILVA SSU rRNA database (release 138.1; threshold set at 0.8). Sequences classified as chloroplasts, mitochondria, or non-bacterial were removed. Data were normalized across samples based on the sample with the smallest sequence count.

### 2.5 Statistical analysis

All statistical and diversity analyses were conducted using R software (Version 4.0.3) and QIIME. For α-diversity, the Chao1 index (reflecting species richness) and Shannon index (reflecting species diversity) were calculated. Differences between two groups were assessed using the Wilcoxon rank-sum test, while comparisons involving multiple groups were performed using the Kruskal–Wallis test. These non-parametric approaches were selected because they are robust to imbalances in subgroup sizes (e.g., Control vs. Benign) and do not require the assumption of normal distribution. To assess β-diversity, differences in microbial community structure were measured using the unweighted UniFrac distance matrix and visualized via principal coordinate analysis (PCoA). Statistical significance of group separation was evaluated using ANOSIM or Adonis (PERMANOVA). Given the notable imbalance in smoking status and the potential heterogeneity in pathological type between early-stage and advanced-stage lung cancer, we applied multivariable approaches to adjust for these potential confounders. Specifically, multivariable PERMANOVA (Adonis) was used for β-diversity analyses, and MaAsLin2 (Microbiome Multivariable Association with Linear Models) was employed for differential abundance testing, with age, sex, smoking status, and pathological type included as covariates. For pathological type, tumors were categorized as adenocarcinoma versus non-adenocarcinoma (including squamous cell carcinoma and other histologies) to avoid model overfitting given the limited sample size. In addition, a sensitivity analysis restricted to the adenocarcinoma-only population was performed to evaluate whether the stage-related microbial differences remained detectable after minimizing histological heterogeneity. These analyses were designed to test whether the observed microbial signatures primarily reflected tumor progression rather than differences in histological composition.

Differential Species Screening—LEfSe (Linear discriminant analysis Effect Size): LEfSe was used as an exploratory, unadjusted method to identify candidate biomarkers across different groups. A linear discriminant analysis (LDA) score threshold (Log LDA Score) > 3.5 and a Kruskal-Wallis test *P* < 0.05 were considered statistically significant. Because LEfSe does not support covariate adjustment, all covariate-adjusted differential abundance analyses were performed separately using MaAsLin2, with age, sex, smoking status, and pathological type included as fixed-effect covariates.

Trend Analysis: For specific bacterial genera (e.g., *Enterobacterales*, *Roseburia*), their relative abundance trends across the disease spectrum (healthy → benign → early-stage → advanced-stage) were visualized using boxplots or heatmaps. The significance of trends was assessed using the Jonckheere-Terpstra trend test or linear regression models. Heatmaps were generated based on Z-score normalized relative abundance data.

## 3 Results

### 3.1 Clinical characteristics of the study participants

A total of 111 participants meeting the inclusion and exclusion criteria were enrolled in this study. Based on clinical diagnosis and pathological findings, participants were categorized into the non-tumor group (Group N, *n* = 54) and the lung cancer patient group (Group L, *n* = 57). The mean age of Group N was 60.22 years ( ± 10.43), while that of Group L was 63.42 years ( ± 11.80) (*P* = 0.082). The mean BMI in the non-tumor group was 24.82 kg/m^2^ ( ± 3.92), compared to 24.45 kg/m^2^ ( ± 3.95) in the lung cancer group, showing no statistically significant difference (*P* = 0.615). The proportion of smokers was significantly higher in the lung cancer group than in the non-tumor group [49.1% (28/57) vs. 22.2% (12/54); *P* = 0.003]. Although this disparity reflects the well-established link between smoking and lung cancer, multivariable analyses adjusting for smoking status confirmed that the observed core microbial shifts were independently associated with the disease state. Conversely, the proportion of alcohol consumers did not differ significantly between the two groups [non-tumor: 37.0% (20/54); lung cancer: 40.4% (23/57); *P* = 0.723).

The non-tumor group was further subdivided into healthy controls (Group C, *n* = 36) and individuals with benign pulmonary nodules (Group B, *n* = 18). The lung cancer group was subdivided into treatment-naive early-stage patients (Group EL, *n* = 20) and advanced-stage patients (Group AL, *n* = 37). The EL group consisted solely of patients with stage I-II disease according to the AJCC 8th edition. The detailed TNM distribution was as follows: T1 (*n* = 11), T2 (*n* = 7), T3 (*n* = 2); N0 (*n* = 17), N1 (*n* = 3); M0 (*n* = 20). Notably, no patient had T4, N2-3, or M1 disease, confirming that all cases were within the defined early-stage range. In the AL group, T category was T1-2 in 9 patients, T3 in 14 patients, and T4 in 14 patients; nodal status was N0-1 in 9 patients and N2-3 in 28 patients. With respect to distant spread, 22 of 37 advanced-stage patients had M1 disease, most commonly involving bone (*n* = 9), brain (*n* = 6), adrenal gland (*n* = 4), and liver (*n* = 3), whereas no intestinal metastasis was identified in any patient. These data indicate that the gut microbiota alterations observed in this cohort were unlikely to be explained by direct intestinal tumor involvement alone. At the same time, because the advanced-stage group contained a relatively higher proportion of squamous cell carcinoma and other non-adenocarcinoma histologies, additional analyses controlling for pathological type were performed to verify that the stage-dependent microbial differences were not solely driven by histological imbalance. Demographic characteristics, clinicopathological features, and metastatic characteristics for each group are summarized in Table 1. With respect to histological composition, adenocarcinoma remained the predominant subtype in both the early-stage and advanced-stage groups, accounting for 80.0% (16/20) and 51.4% (19/37), respectively, whereas squamous cell carcinoma accounted for 10.0% (2/20) and 21.6% (8/37), and other histologies including small cell and neuroendocrine carcinomas accounted for 10.0% (2/20) and 27.0% (10/37), respectively. Although the overall distribution of pathological type did not reach statistical significance between the EL and AL groups (χ^2^ = 4.523, *P* = 0.1042), the advanced-stage group showed a relatively higher proportion of non-adenocarcinoma histologies, which was therefore considered in subsequent sensitivity and multivariable analyses. Notably, all participants were recruited from the same metropolitan area (Beijing, China) and shared a relatively consistent Northern Chinese dietary pattern; this geographic and dietary homogeneity helps minimize the confounding effects of large-scale environmental variation on the oral and gut microbiota. In addition, because no intestinal metastasis was present in the enrolled cohort, the stage-related gut microbial differences identified in this study are more likely to reflect systemic effects associated with tumor progression and metastatic burden rather than direct seeding of tumor cells into the intestinal tract.

**TABLE 1:** Demographic, clinicopathological, and metastatic characteristics of the study groups.

Index	C (healthy control) (*n* = 36)	B (benign nodule) (*n* = 18)	EL (early-stage lung cancer) (*n* = 20)	AL (advanced-stage lung cancer) (*n* = 37)	Statistic	*P*-value
Sex (Male), n (%)	20 (55.6%)	6 (33.3%)	8 (40.0%)	27 (73.0%)	χ^2^ = 10.064	0.018
Age (years)	60.28 ± 11.70	60.11 ± 8.24	62.60 ± 12.36	64.11 ± 11.30	F = 0.908	0.4396
BMI (kg/m^2^)	24.51 ± 3.82	25.15 ± 3.94	23.95 ± 4.17	24.40 ± 3.47	F = 0.498	0.684
Smoking, n (%)	10 (27.8%)	2 (11.1%)	2 (10.0%)	26 (70.3%)	χ^2^ = 30.611	1.03 × 10^−6^
Alcohol consumption, n (%)	16 (44.4%)	4 (22.2%)	4 (20.0%)	19 (51.4%)	χ^2^ = 8.002	0.046
AJCC stage, n (%)	–	–	I–II: 20 (100.0%)	III–IV: 37 (100.0%)	–	–
Pathological type, n (%)	–	–			χ^2^ = 4.523	0.1042
Adenocarcinoma	–	–	16 (80.0%)	19 (51.4%)		
Squamous cell carcinoma	–	–	2 (10.0%)	8 (21.6%)		
Other (e.g., small cell, neuroendocrine)	–	–	2 (10.0%)	10 (27.0%)		
T category distribution, n (%)	–	–			–	–
T1	–	–	11 (55.0%)	3 (8.1%)		
T2	–	–	7 (35.0%)	6 (16.2%)		
T3	–	–	2 (10.0%)	14 (37.8%)		
T4	–	–	0 (0.0%)	14 (37.8%)		
N category distribution, n (%)	–	–			–	–
N0	–	–	17 (85.0%)	4 (10.8%)		
N1	–	–	3 (15.0%)	5 (13.5%)		
N2	–	–	0 (0.0%)	16 (43.2%)		
N3	–	–	0 (0.0%)	12 (32.4%)		
M category distribution, n (%)	–	–			–	–
M0	–	–	20 (100.0%)	15 (40.5%)		
M1	–	–	0 (0.0%)	22 (59.5%)		
Presence of symptoms (Symptomatic), n (%)	–	–	6 (30.0%)	32 (86.5%)	Fisher’s exact test	2.82 × 10^−5^
Intestinal metastasis, n (%)	0 (0.0%)	0 (0.0%)	0 (0.0%)	0 (0.0%)	–	–
Other distant metastasis sites in AL, n	–	–	–	Bone, 9; Brain, 6; Adrenal gland, 4; Liver, 3	–	–

Early-stage lung cancer (EL) was strictly defined as AJCC 8th edition stage I–II disease, and advanced-stage lung cancer (AL) was defined as stage III–IV disease. T, N, and M categories are presented as descriptive distributions rather than grouping criteria.

### 3.2 Overview of microbial composition

#### 3.2.1 Overview of overall taxon abundance

**FIGURE 2:**
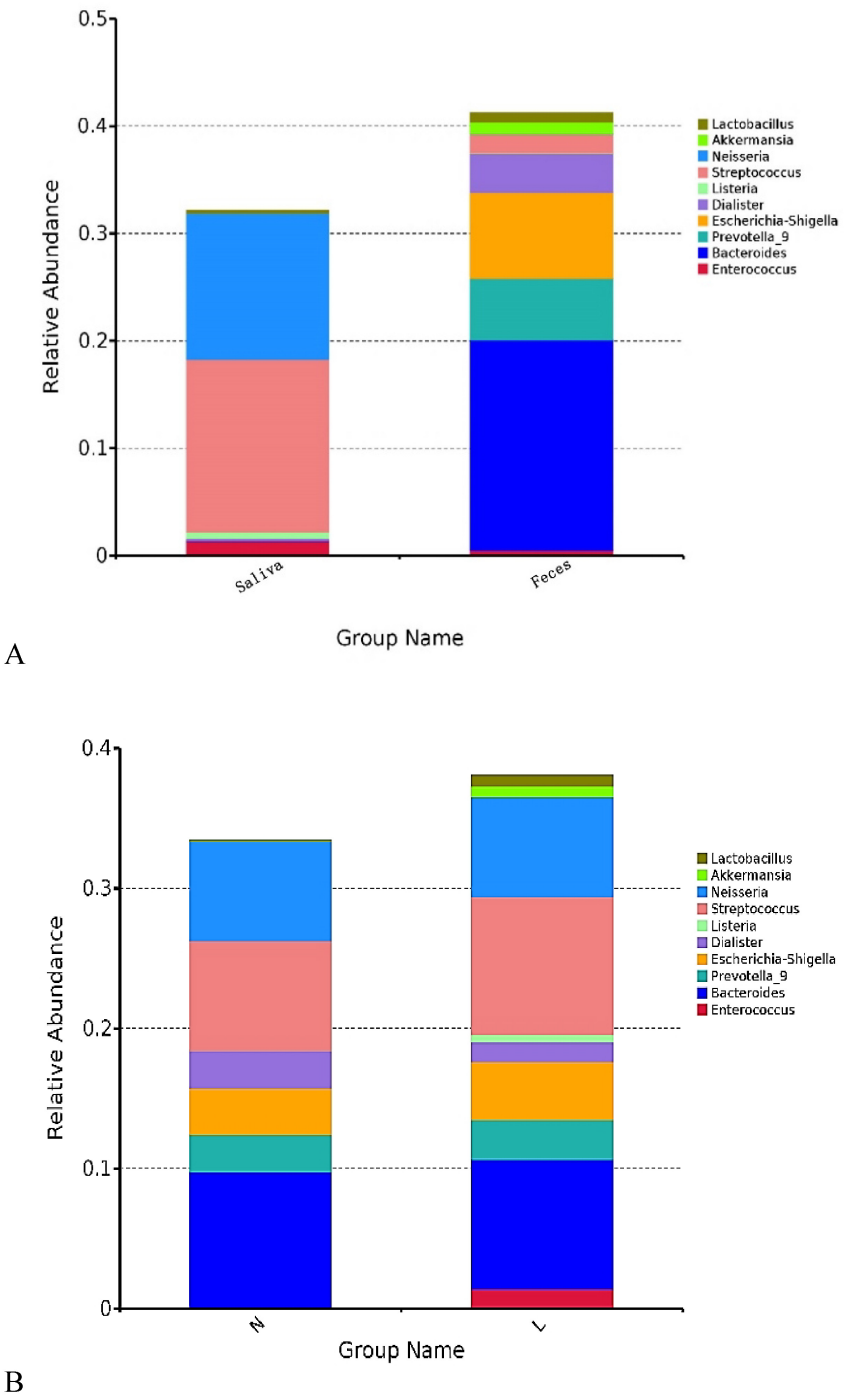
Overview of microbial community composition at the genus level across the study cohort. **(A)** Comparison of the distribution of the top 10 most abundant genera between saliva and fecal samples. Saliva samples were dominated by *Neisseria* and *Streptococcus*, while fecal samples were dominated by *Bacteroides* and *Escherichia-Shigella*. **(B)** Comparison of microbial community composition between the non-tumor group (Group N) and the lung cancer patient group (Group L). Compared to Group N, visible changes were observed in the relative abundance of genera such as *Enterococcus* in Group L. The y-axis represents relative abundance, with different colors representing different genera.

Statistical analysis of relative abundance at the genus level for all samples revealed distinct distribution patterns of dominant microbiota, varying by sampling site (oral vs. gut) and health status (non-tumor vs. lung cancer). As shown in Figure 2A, saliva and fecal samples harbored distinctly different core microbiota, reflecting the colonization preferences of these two distinct ecological niches. In saliva samples, the microbial community was predominantly composed of Firmicutes and Proteobacteria. The most relatively abundant genera were *Streptococcus* and *Neisseria*, which absolutely dominated the salivary microbiota. Additionally, *Enterococcus*, *Listeria*, and *Lactobacillus* also showed high enrichment in saliva.

In fecal samples, the community structure was dominated by anaerobic bacteria. *Bacteroides* was the most abundant dominant genus, followed by *Escherichia-Shigella* and *Prevotella_9*. *Dialister* also constituted a certain proportion of the fecal microbiota. Notably, although *Streptococcus* was present in feces, its relative abundance was significantly lower than in saliva.

After pooling sample types, further comparison of microbial composition between Group N and Group L (Figure 2B) showed that while the two groups shared most core genera, discernible shifts occurred in the relative abundance of specific taxa. Compared with Group N, the microbial community in Group L showed a relative expansion of *Enterococcus* and *Streptococcus*, a reduction in *Neisseria*, and a tendency toward enrichment of *Akkermansia* (Figure 2B). These taxonomic shifts preliminarily suggest microbial dysbiosis associated with lung cancer. These fluctuations in the abundance of dominant genera preliminarily suggest dysbiosis of the microbial community structure associated with lung cancer.

#### 3.2.2 α-diversity analysis

α-diversity primarily reflects the species richness and diversity within a single microbial community. This study used the Chao1 index to assess species richness (i.e., the number of species within a community) and the Shannon index to comprehensively evaluate community diversity (considering both species richness and evenness).

As shown in Table 2, for saliva samples (Group S), no significant differences in α-diversity indices were observed between the non-tumor group (S.N) and lung cancer patients (S.L). The median Chao1 indices were comparable between the two groups (*P* = 0.111). The distribution ranges of the Shannon index also overlapped considerably, with no statistically significant difference (*P* = 0.714). These findings suggest that, following lung cancer onset, the overall richness and diversity structure of the oral microbiota (proximal respiratory tract entry) remained relatively stable, without dramatic collapse or reorganization.

**TABLE 2:** Comparison of oral and gut microbiota α-diversity between the non-tumor group (Group N) and lung cancer patients (Group L).

Group	Median	IQR (interquartile range)	IQR Width (Q3−Q1)	Lower whisker—upper whisker	Outliers	Statistical test
Saliva/Chao1 Index						
S.N	∼300	∼260–340	∼80	∼150–450	1 low outlier (∼110); 1 high outlier (∼570)	Between-group comparison: *P* = 0.111
S.L	∼320–330	∼260–380	∼120	∼80–520	Multiple high outliers (∼600–680)	
Saliva/Shannon index						
S.N	∼5.7	∼5.3–6.1	∼0.8	∼4.1–6.9	None	Between-group comparison: *P* = 0.714
S.L	∼5.7	∼5.2–6.1	∼0.9	∼4.0–6.9	Multiple low outliers (∼3.7, ∼3.5, ∼2.2, ∼1.2)	
Feces/Chao1 index						
F.N	∼250	∼200–330	∼130	∼50–450	1 low outlier (∼50)	Between-group comparison: *P* = 0.009
F.L	∼450	∼350–550	∼200	∼200–1000	Multiple high outliers (∼650, ∼700, ∼800)	
Feces/Shannon index						
F.N	∼5.9	∼5.3–6.3	∼1.0	∼4.0–7.5	Multiple low outliers (∼3.5, ∼3.7, ∼4.1)	Between-group comparison: *P* = 0.294
F.L	∼6.2	∼5.5–6.6	∼1.1	∼4.0–7.0		

In contrast to saliva samples, α-diversity analysis of fecal samples (Group F) revealed significant differences between groups. The Chao1 index of the gut microbiota in Group L patients was significantly higher than that in Group N (*P* = 0.009), indicating a greater variety of microorganisms (increased OTU count) within the intestines of lung cancer patients. This phenomenon might be associated with changes in the intestinal microenvironment under disease conditions, leading to abnormal proliferation of certain non-dominant opportunistic pathogens or rare species. Despite the increased richness, the Shannon index showed no statistically significant difference between the two groups (*P* = 0.294), suggesting that the newly emerged species likely had low abundance and were insufficient to alter the overall community evenness significantly.

#### 3.2.3 β-diversity comparison (PCoA analysis)

Differences in microbial community structure between the non-tumor group (Group N) and lung cancer patients (Group L) were visualized and quantified using principal coordinate analysis (PCoA) based on the Unweighted UniFrac distance matrix, along with statistical testing.

**FIGURE 3:**
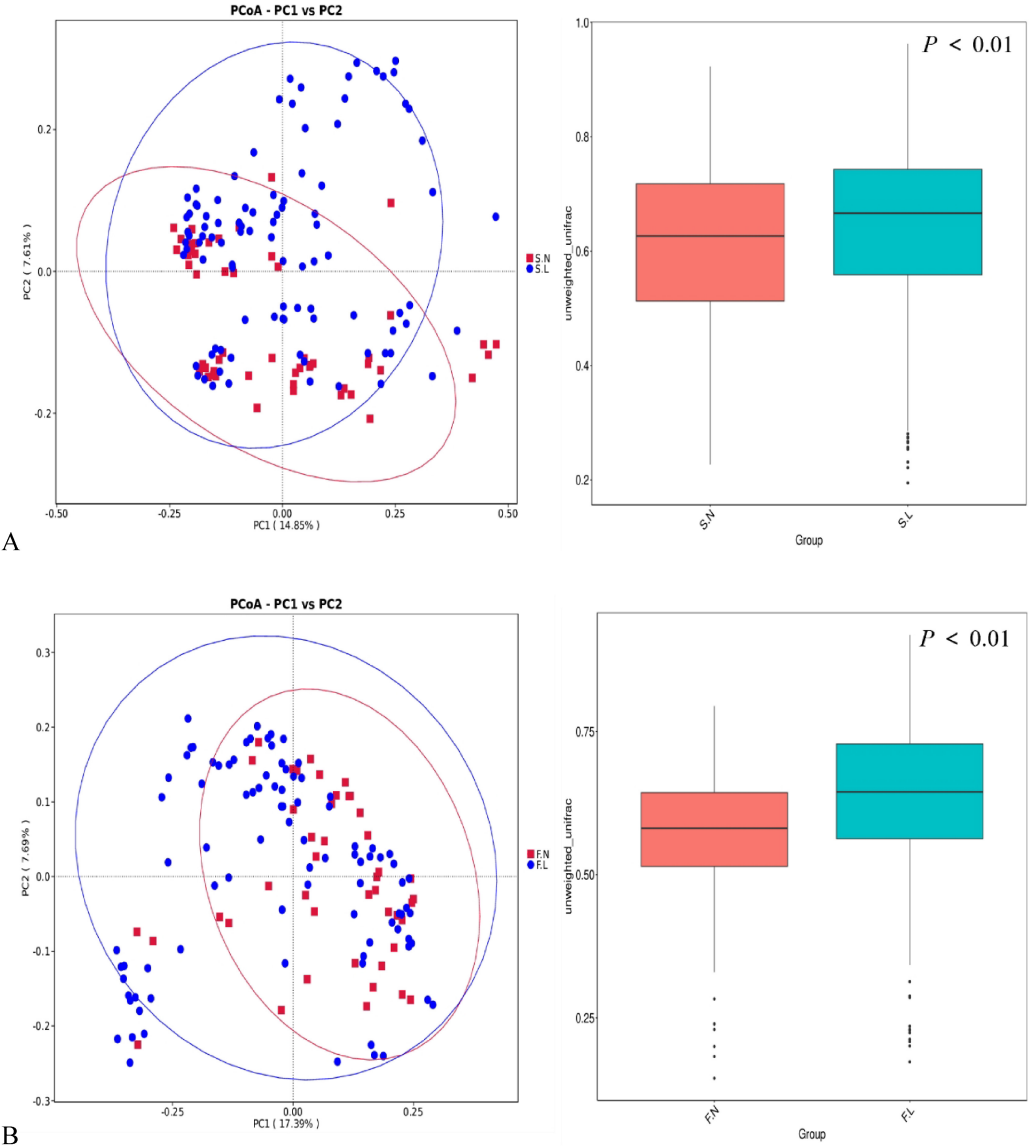
β-diversity principal coordinate analysis (PCoA) of non-tumor (N) and lung cancer (L) groups. **(A)** PCoA plot of saliva samples. Red squares represent the non-tumor group (S.N), and blue dots represent the lung cancer group (S.L). **(B)** PCoA plot of fecal samples. The x-axis and y-axis represent the first principal coordinate (PC1) and the second principal coordinate (PC2), respectively, with the percentage of variation explained shown in parentheses.

PCoA analysis of saliva samples (Figure 3A) showed a trend toward separation of the community centroids between the non-tumor group (S.N, red) and the lung cancer group (S.L, blue), despite some overlap in spatial distribution. Quantifying this difference, the Unweighted UniFrac distance (median ≈ 0.67) within the saliva microbiota of the L group was significantly higher than that of the N group (median ≈ 0.63) (*P* < 0.01), suggesting a significant structural shift in the oral microbial composition of lung cancer patients, characterized by a broader or more unique set of species.

PCoA analysis of fecal samples demonstrated a clearer separation pattern between groups (Figure 3B). PC1 explained 17.39% of the variation, and PC2 explained 7.69%. Although inter-individual heterogeneity existed, the distribution areas of the non-tumor group (F.N) and the lung cancer group (F.L) were distinguishable along the axes. Further analysis confirmed that the β-diversity distance (median ≈ 0.64) of the gut microbiota in lung cancer patients was significantly greater than that in the non-tumor group (median ≈ 0.56) (*P* < 0.01). This indicates profound remodeling (dysbiosis) of the gut ecosystem in lung cancer patients compared to healthy or benign states, with the species composition fundamentally differing from that of non-tumor individuals.

### 3.3 Identification of differential species between groups (LEfSe analysis)

#### 3.3.1 Saliva biomarkers

**FIGURE 4:**
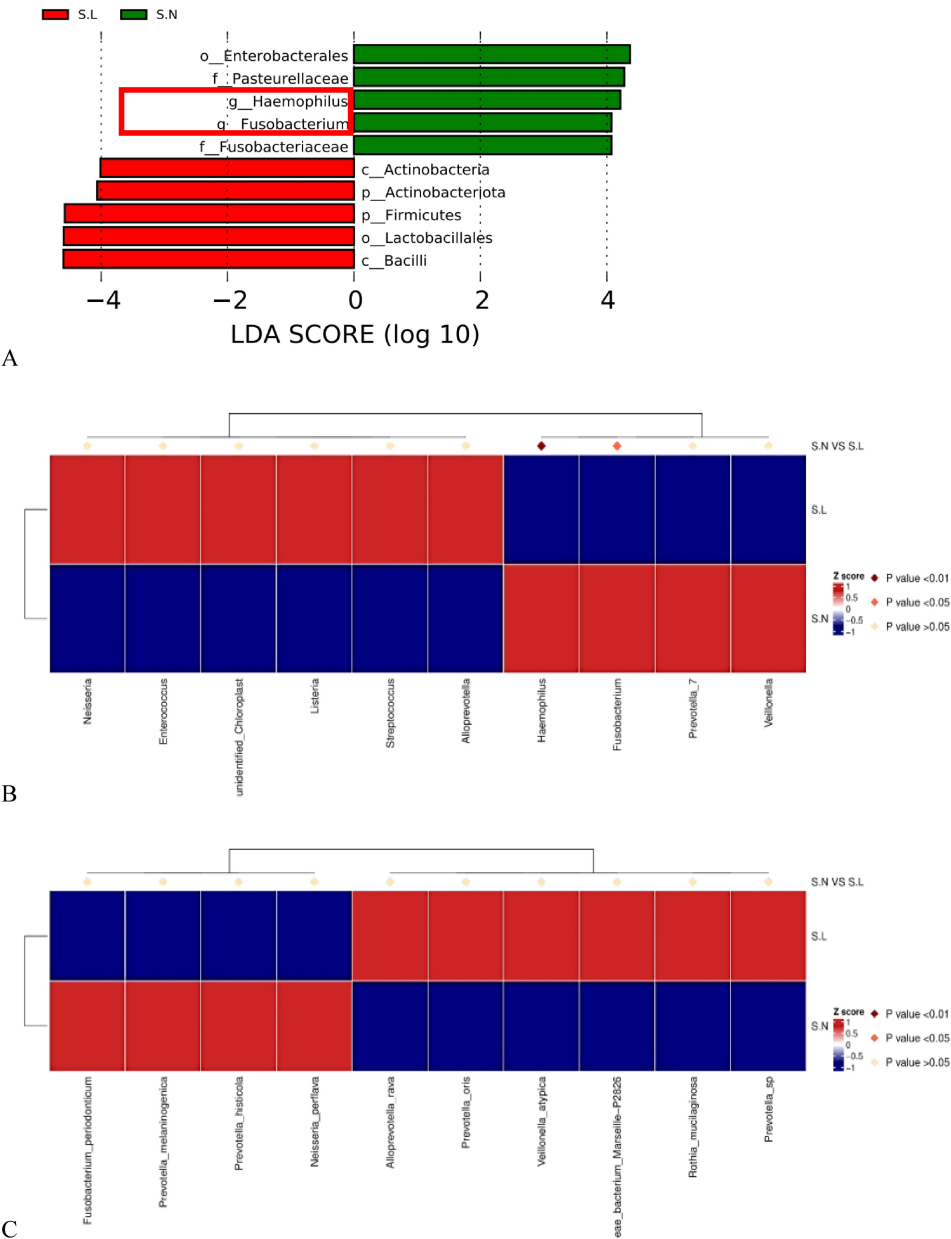
Differential species analysis in saliva samples between non-tumor (S.N) and lung cancer (S.L) groups based on LEfSe. LEfSe results are unadjusted; covariate-adjusted differential abundance results obtained using MaAsLin2 are provided in Supplementary Table 1. **(A)** LEfSe analysis histogram for saliva samples. The x-axis represents the LDA Score (Log10), reflecting the contribution of differential taxa to distinguishing between groups. Green bars indicate taxa enriched in the non-tumor group (S.N) (e.g., *Haemophilus*, *Fusobacterium*); red bars indicate taxa enriched in the lung cancer group (S.L) (e.g., Actinobacteriota, Firmicutes). **(B)** Heatmap of differential species abundance at the genus level in saliva samples. Red represents high relative abundance, blue represents low relative abundance. Diamonds above indicate the statistical significance of inter-group differences (dark red: *P* < 0.01; light red: *P* < 0.05). Red boxes highlight *Haemophilus* and *Fusobacterium*, which were significantly enriched in the non-tumor group (S.N). **(C)** Heatmap of differential species abundance at the species level in saliva samples. It illustrates features such as *Fusobacterium periodonticum* enriched in the S.N group and *Rothia mucilaginosa* enriched in the S.L group.

LEfSe analysis results (Figure 4A) revealed differences in the salivary microbiota between lung cancer patients and non-tumor individuals across multiple taxonomic levels, including phylum, class, order, family, and genus.

Characteristic taxa of the non-tumor group (S.N): As indicated by the green bars in Figure 4A, the non-tumor group was significantly enriched in *Pasteurellaceae* (under Proteobacteria) and its genus *Haemophilus*, as well as *Fusobacterium* (under Fusobacteriaceae). In the genus-level heatmap (Figure 4B), marked by red boxes, *Haemophilus* and *Fusobacterium* exhibited high abundance (red) in the non-tumor group (S.N, bottom row) but were significantly decreased (blue) in the lung cancer group (S.L, top row), with statistical tests showing highly significant differences (*P* < 0.01, dark red diamonds). This suggests a negative correlation between the abundance of these two oral commensal bacteria and lung cancer development.

Characteristic taxa of lung cancer patients (S.L): As shown by the red bars in Figure 4A, the salivary microbiota of lung cancer patients underwent a significant structural shift, characterized by abnormal enrichment of Actinobacteriota and Firmicutes. At finer taxonomic levels, Actinobacteria, Bacilli, and Lactobacillales were all identified as characteristic markers of the lung cancer group. This finding suggests that alterations in the oral microenvironment of lung cancer patients might favor the colonization and proliferation of Gram-positive bacteria (Actinobacteria and Firmicutes).

Further analysis at the species level (Figure 4C) corroborated these findings. The non-tumor group was significantly enriched in Fusobacterium periodonticum and Prevotella melaninogenica, whereas the lung cancer group exhibited high abundance of species such as Prevotella oris, Veillonella atypica, and Rothia mucilaginosa. It should be noted that the taxa shown in Figure 4 were identified by LEfSe and therefore represent unadjusted results; covariate-adjusted associations are provided separately by MaAsLin2 (Supplementary Table 1).

#### 3.3.2 Gut biomarkers

**FIGURE 5:**
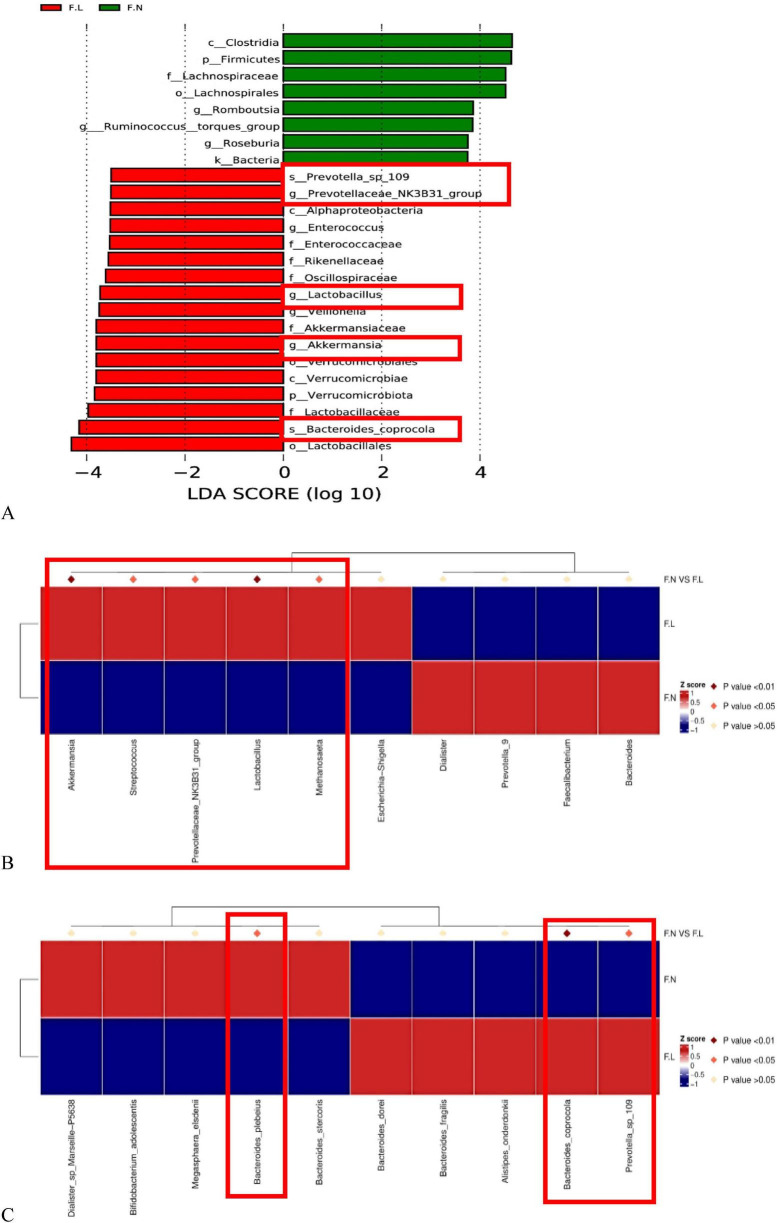
Differential species analysis in fecal samples between non-tumor (F.N) and lung cancer (F.L) groups based on LEfSe. LEfSe results are unadjusted; covariate-adjusted differential abundance results obtained using MaAsLin2 are provided in Supplementary Table 1. **(A)** LEfSe analysis histogram for fecal samples (LDA Score > 3.5). Green bars represent taxa enriched in the non-tumor group (F.N) (including Firmicutes, *Roseburia*, *Ruminococcus*); red bars represent taxa enriched in the lung cancer group (F.L) (including *Bacteroides coprocola*, *Lactobacillus*, *Akkermansia*). **(B)** Heatmap of differential species abundance at the genus level in fecal samples. Red boxes indicate that *Akkermansia*, *Streptococcus*, *Lactobacillus*, and *Prevotellaceae_NK3B31_group* were significantly enriched in the lung cancer group (F.L) (dark red diamonds above represent *P* < 0.01). **(C)** Heatmap of differential species abundance at the species level in fecal samples. The left red box shows *Bacteroides plebeius* enriched in the non-tumor group; the right red box shows *Bacteroides coprocola* and *Prevotella* sp. 109 significantly enriched in the lung cancer group (*P* < 0.01).

LEfSe analysis of fecal samples (Figure 5A) further revealed specific alterations in the gut microbiota structure of lung cancer patients. Similar to saliva samples, significant separation in gut microbial composition was observed between non-tumor individuals and lung cancer patients.

Characteristic taxa of the non-tumor group (F.N): As shown by the green bars in Figure 5A, the gut microbiota of the non-tumor group was primarily dominated by Firmicutes and its class Clostridia. At the genus level, this group was significantly enriched in *Roseburia*, *Ruminococcus_torques_group*, and *Romboutsia*. Many of these genera are beneficial commensal bacteria in the human gut. Notably, *Roseburia* and *Ruminococcus* are well-known producers of short-chain fatty acids (SCFAs, e.g., butyrate), which play crucial roles in maintaining intestinal mucosal barrier function and suppressing inflammation. This suggests that the non-tumor group possesses a healthier gut microbial ecosystem. In contrast, the gut microbiota of lung cancer patients (F.L, red bars) exhibited a trend toward increased abundance of potentially pathogenic or oral-origin bacteria.

Integrating LEfSe results with the genus-level heatmap (Figure 5B), the lung cancer group showed significant enrichment of *Streptococcus*, *Lactobacillus*, *Veillonella*, and *Akkermansia*. Notably, *Streptococcus*, *Veillonella*, and *Lactobacillus* are typically resident bacteria of the oral cavity or upper respiratory tract. Their abnormal enrichment in the gut may imply ectopic colonization of “oral-gut” bacteria, providing direct evidence for gut-lung axis microbial interaction in lung cancer patients, whereby oral-origin microbes potentially bypass gastric barriers to influence pulmonary health via the common mucosal immune network.

Although the differences at the overall genus level were complex, species-level analysis (Figure 5C) revealed significant differentiation within the genus *Bacteroides*. The lung cancer group specifically enriched *Bacteroides coprocola* (*P* < 0.01), while the non-tumor group enriched *Bacteroides plebeius*. Additionally, *Prevotella* sp. 109 also showed significantly high abundance in the lung cancer group. Thus, the gut microbiota signature in lung cancer patients is characterized by depletion of beneficial butyrate-producing bacteria (e.g., Roseburia) and abnormal proliferation of oral-origin bacteria (e.g., Streptococcus) and specific Bacteroides species like B. coprocola. As with the salivary LEfSe results, the taxa displayed in Figure 5 represent unadjusted findings, whereas the covariate-adjusted differential abundance results obtained using MaAsLin2 are summarized in Supplementary Table 1.

#### 3.3.3 Independence of microbial signatures from smoking status

Given the significant difference in smoking rates between the lung cancer and non-tumor groups, we performed covariate-adjusted differential abundance analysis using MaAsLin2. After adjustment for age, sex, smoking status, and pathological type, the depletion of salivary Haemophilus (adjusted *P* = 0.021) and the enrichment of gut Bacteroides coprocola (adjusted *P* = 0.006) remained significantly associated with lung cancer. These adjusted results are distinct from the exploratory LEfSe results shown in Figures 4, 5 and are summarized in Supplementary Table 1. This indicates that the observed oral-gut dysbiosis is an independent biological characteristic of the disease state rather than a mere reflection of smoking-induced alterations.

#### 3.3.4 Sensitivity analysis for histological composition

**FIGURE 6:**
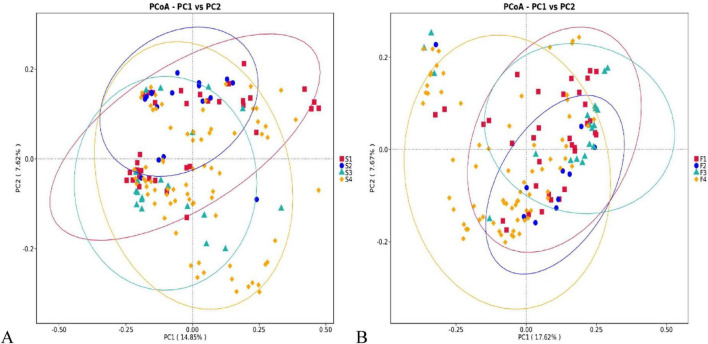
β-diversity distribution characteristics of saliva and feces across the four subgroups. **(A)** PCoA plot of saliva samples from the four subgroups (S1: healthy control, red; S2: benign nodule, blue; S3: early-stage lung cancer, green; S4: advanced-stage lung cancer, orange). Ellipses represent confidence intervals for each group. **(B)** PCoA plot of fecal samples from the four subgroups (F1-F4, corresponding colors). Ellipses represent confidence intervals for each group. The separation trend between early-stage and advanced-stage lung cancer remained evident in sensitivity analyses restricted to adenocarcinoma cases and in multivariable models adjusting for age, sex, smoking status, and pathological type.

**FIGURE 7:**
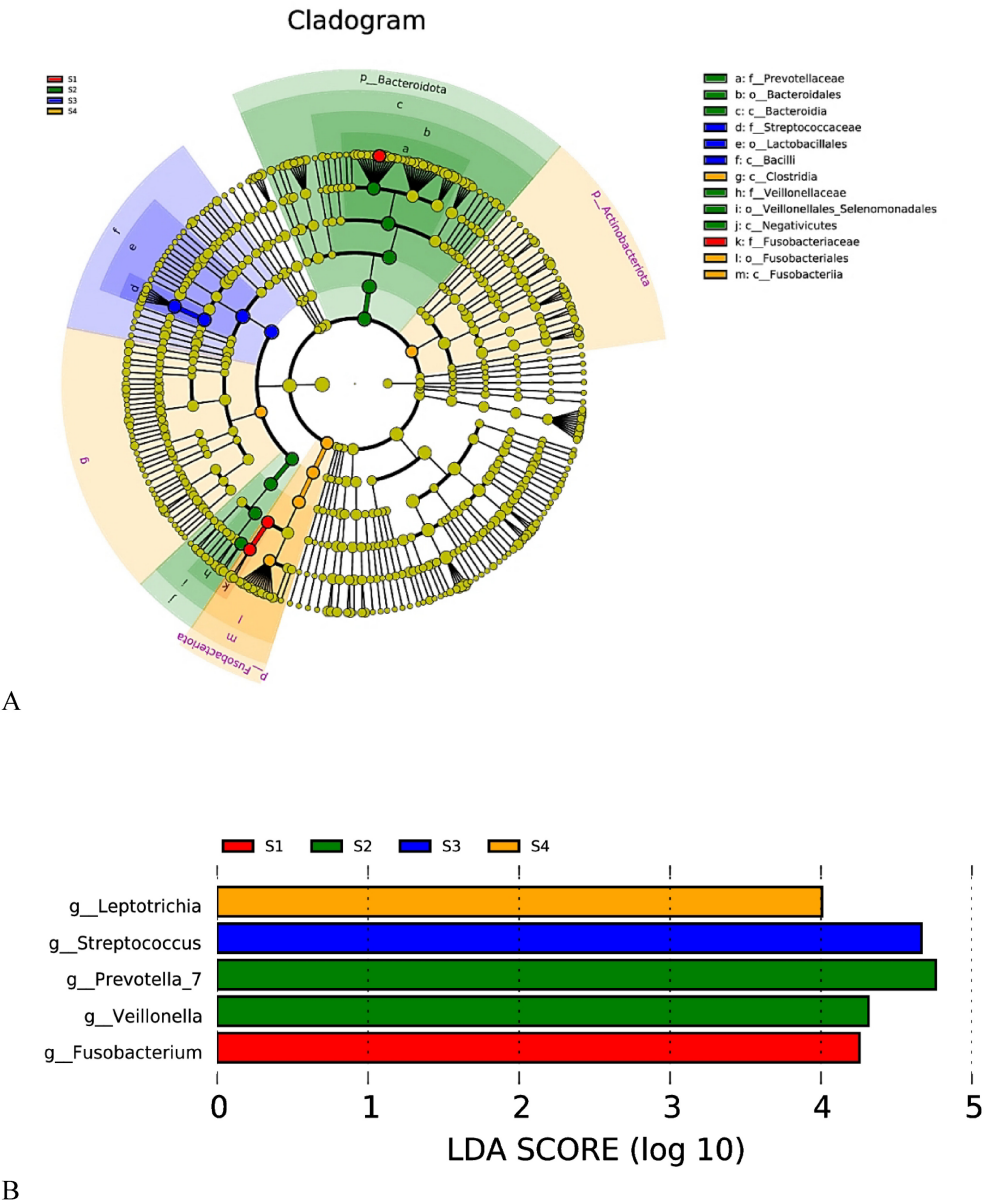
LEfSe analysis of differential taxa in saliva samples (S1–S4): cladogram and LDA effect size bar plot. LEfSe results are unadjusted; corresponding covariate-adjusted associations evaluated by MaAsLin2 are summarized in Supplementary Table 1. **(A)** Cladogram: The circles radiating from inner to outer layers represent taxonomic levels from phylum to genus/species. Nodes in different colors represent taxa significantly enriched in the corresponding group; yellow nodes indicate non-significant differences. The sector-shaped background highlights the phylum level (e.g., Bacteroidota, Firmicutes, Fusobacteriota) of the main differential branches. **(B)** LDA score (log10) bar plot: Displays the main discriminatory genera and their effect sizes. Longer bars indicate a greater contribution of the taxon to inter-group differences. Results show: S1 enriched *Fusobacterium*; S2 enriched *Prevotella_7* and *Veillonella*; S3 enriched *Streptococcus*; S4 enriched *Leptotrichia*.

**FIGURE 8:**
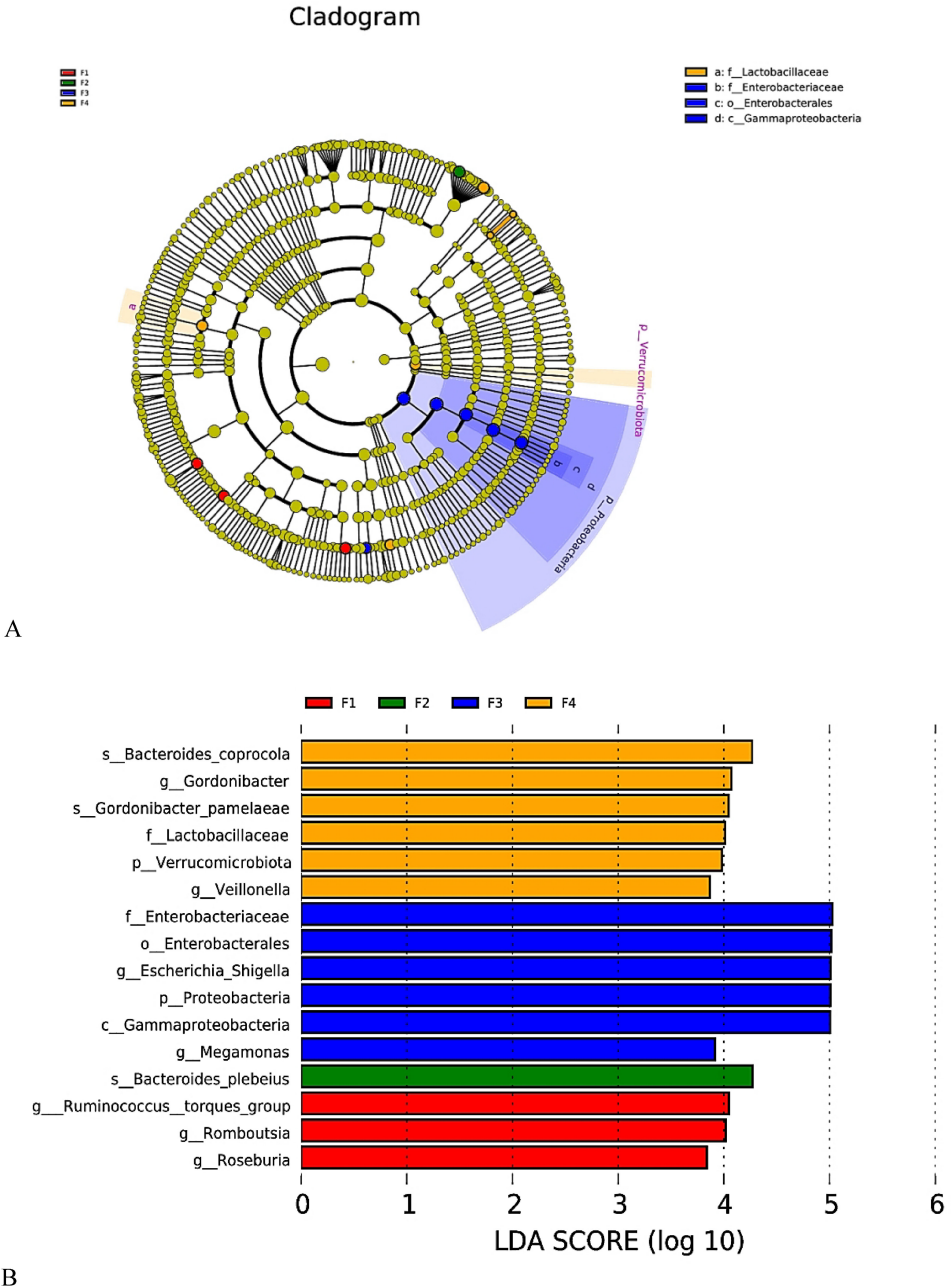
Differential gut microbiota biomarkers across different groups identified by LEfSe analysis. LEfSe results are unadjusted; corresponding covariate-adjusted associations evaluated by MaAsLin2 are summarized in Supplementary Table 1.

Because the relative proportions of adenocarcinoma, squamous cell carcinoma, and other histological subtypes differed between the early-stage and advanced-stage groups, we performed additional analyses to evaluate whether the observed microbial differences were confounded by histology. First, pathological type was incorporated as a covariate in multivariable PERMANOVA and MaAsLin2 models together with age, sex, and smoking status. After adjustment, the overall stage-related separation of fecal microbial communities remained significant (adjusted PERMANOVA, *P* = 0.021). In addition, MaAsLin2 confirmed that key stage-related taxa remained independently associated with disease progression, including salivary Rothia mucilaginosa (adjusted *P* = 0.018) and fecal Bacteroides coprocola (adjusted *P* = 0.006). These covariate-adjusted results are summarized in Supplementary Table 1, whereas the taxa shown in Figures 7, 8 are exploratory LEfSe findings and should be interpreted as unadjusted. Second, a sensitivity analysis restricted to adenocarcinoma cases only (EL: *n* = 16; AL: *n* = 19) yielded directionally consistent results, with preservation of the early-versus-advanced separation trend in β-diversity (*P* = 0.034) and similar stage-associated abundance shifts of the major candidate taxa. Collectively, these findings indicate that the microbial differences highlighted in Figure 6 are not merely attributable to histological composition imbalance, but are more likely associated with tumor progression itself.

### 3.4 Comparative analysis across four subgroups

To further investigate the dynamic evolution of microbial communities during lung cancer development and progression, participants were stratified into four subgroups for in-depth comparison: healthy control group (Group 1), benign pulmonary nodule group (Group 2), treatment-naive early-stage lung cancer group (Group 3), and treatment-naive advanced-stage lung cancer group (Group 4).

#### 3.4.1 Comparison of α-diversity

As shown in Table 3, Chao1 and Shannon indices in saliva and feces differed significantly across the four subgroups (*P* < 0.05).

**TABLE 3:** α-diversity of saliva and fecal samples across the four subgroups (healthy, benign, early-stage cancer, advanced-stage cancer).

Group	Median	IQR (Interquartile range)	Range	Outliers	Statistical test
Saliva/Chao1 Index					*H* = 14.722, *P* = 0.002
S1	∼320	∼265–355	∼160–440	None	
S2	∼270	∼190–305	∼100–360	1 outlier (∼550)	
S3	∼230	∼165–345	∼60–480	1 extremely high outlier (∼680)	
S4	∼340	∼275–385	∼180–540	Multiple outliers (580–650 range)	
Saliva/Shannon Index					*H* = 14.558, *P* < 0.01
S1	∼5.7	∼5.3–6.2	∼4.2–6.8	1 outlier (∼4.1)	
S2	∼5.4	∼5.0–6.1	∼4.1–6.8	None	
S3	∼4.6	∼4.2–5.7	∼2.2–6.3	1 extremely low outlier (∼1.5)	
S4	∼5.8	∼5.3–6.2	∼4.2–7.3	3 outliers (∼3.9–4.1)	
Feces/Chao1 Index					H = 15.840, P = 0.001
F1	∼210	∼170–250	∼120–320	∼430–480 (approximately 2 high-value points)	
F2	∼250	∼220–300	∼170–330	∼490 (approximately 1 high-value point)	
F3	∼160	∼130–240	∼100–430	∼490–550 (approximately 2 high-value points)	
F4	∼300	∼220–450	∼80–780	∼820–900 (approximately 3) + ∼1200, ∼1400 (higher outliers)	
Feces/Shannon Index					*H* = 66.184, *P* < 0.001
F1	∼4.5	∼4.0–5.5	∼4.0–5.5	1 outlier (∼4.0)	
F2	∼4.7	∼4.2–5.5	∼4.2–5.5	None	
F3	∼6.5	∼5.0–7.5	∼5.0–7.5	None	
F4	∼7.5	∼5.5–8.5	∼5.5–8.5	None	

* Table content omitted as per request, showing median, IQR, range, outliers, and statistical test results (*H-* and *P-*values) for Chao1 and Shannon indices in saliva and feces for groups S1–S4 and F1–F4.

The salivary Chao1 index was significantly higher in the advanced lung cancer group (S4) compared to the benign nodule group (S2) and early lung cancer group (S3) (S2 vs. S4, *P* < 0.05; S3 vs. S4, *P* < 0.05), suggesting an increase in oral bacterial species richness with increasing tumor burden. The Shannon index in the early lung cancer group (S3) exhibited a marked decreasing trend, reaching the lowest value (S1 vs. S3, *P* < 0.05; S2 vs. S3, *P* < 0.05; S3 vs. S4, *P* < 0.05). This finding suggests that in the early stages of lung cancer, the oral microbiota may undergo a “loss” of diversity or monoclonal proliferation of specific dominant bacteria, only to become complex again in the advanced stage.

The fecal Chao1 index was significantly higher in advanced lung cancer (F4) compared to healthy controls (F1) and early lung cancer (F3) (F1 vs. F4, *P* < 0.05; F3 vs. F4, *P* < 0.05), indicating abnormal species proliferation in the gut of advanced-stage patients. Regarding the Shannon index, the advanced lung cancer group (F4) was significantly higher than the early lung cancer group (F3) (*P* < 0.05). The microbial characteristics of early-stage patients were closer to those of healthy or benign individuals, while advanced-stage patients exhibited significant dysbiosis (manifested as abnormal increases in richness and diversity), potentially related to advanced cachexia and small intestinal bacterial overgrowth (SIBO) secondary to immune dysfunction.

#### 3.4.2 Comparison of β-diversity (PCoA analysis)

Comparison of Chao1 and Shannon indices revealed that the microbial structure in saliva and feces of individuals with benign pulmonary nodules exhibited unique characteristics distinguishing them from healthy controls, early-stage, and advanced-stage lung cancer patients. To further exclude the possibility that these between-stage differences were primarily explained by histological heterogeneity, additional covariate-adjusted and histology-restricted sensitivity analyses were performed for the PCoA-based comparisons. PCoA was employed to further observe the evolutionary dynamics of the salivary microbiota across the four stages, from healthy to advanced cancer. PC1 (14.85%) and PC2 (7.62%) together explained the primary variation in the community. The confidence ellipses of the healthy control group (S1, red) and the benign group (S2, blue) overlapped considerably, and their centroids were relatively close, suggesting that individuals with benign nodules maintain a relatively stable salivary microbial structure. The distribution area of the early lung cancer group (S3, green) showed a shift, and its ellipse was relatively smaller and more concentrated compared to the advanced lung cancer group (S4, orange). As the disease progressed from benign to advanced cancer, the spatial distribution of samples on the coordinate axes gradually became more dispersed, with the advanced group (S4) exhibiting the widest distribution range. This reflects a significant increase in the heterogeneity and complexity of the oral microbial community alongside increasing tumor burden (Figure 6A).

In saliva samples, the benign pulmonary nodule group (S2) demonstrated a relatively stable microbial structure. The Chao1 index (median ∼270) in group S2 was slightly lower than in the healthy group (S1, ∼320) and significantly lower than in the advanced lung cancer group (S4, ∼340, *P* < 0.05). This indicates that although the community structure in benign nodule patients remains intact, total species count has begun to show a slight decreasing trend, contrasting with the “microbial overload” observed in advanced cancer. Furthermore, the median Shannon index in group S2 was approximately 5.4, close to that of the healthy control group (S1, ∼5.7), and both remained at relatively high levels. This contrasts sharply with the significant diversity decline observed in the early lung cancer group (S3, ∼4.6, *P* < 0.05). This suggests that at the benign nodule stage, the oral microbiota has not yet undergone the drastic dysbiosis seen in early-stage lung cancer.

A clearer separation trend was observed along the axes between the non-tumor groups (healthy controls, benign group: F1, F2) and the lung cancer groups (early-stage, advanced-stage: F3, F4). Spatial distribution also differed between the early-stage (F3) and advanced-stage (F4) lung cancer groups, with the advanced-stage group’s confidence ellipse covering the largest area, reflecting greater community heterogeneity, severe dysbiosis, and abnormal microbial proliferation in advanced disease (Figure 6B). Because the histological composition differed to some extent between EL and AL, we further performed multivariable PERMANOVA adjusting for age, sex, smoking status, and pathological type; the overall stage-related separation of fecal microbial communities remained significant (adjusted PERMANOVA, *P* = 0.021). Moreover, in the adenocarcinoma-only sensitivity analysis, the separation trend between early-stage and advanced-stage patients was preserved (*P* = 0.034), indicating that the observed microbial remodeling is primarily associated with tumor progression rather than being solely attributable to imbalances in histological composition.

The Chao1 index in group F2 (median ∼250) was slightly higher than in the healthy control group (F1, ∼210) and the early lung cancer group (F3, ∼160). The Shannon index in group F2 (∼4.7) was marginally higher than in the healthy group (F1, ∼4.5) but far lower than the abnormally high values seen in the advanced lung cancer group (F4). The gut microbiota of benign nodule patients resides in an “intermediate transitional state,” neither exhibiting the species impoverishment of the early lung cancer group (F3) nor the marked microbial disorder and overgrowth of the advanced lung cancer group (F4).

#### 3.4.3 differential species characteristics of salivary microbiota at different disease progression stages (LEfSe analysis)

LEfSe analysis of saliva samples (Saliva, S) revealed distinct characteristic microbial enrichment patterns across different disease stages, suggesting phase-specific remodeling of the oral microbiota during lung cancer progression. A cladogram displayed that differential taxa were mainly distributed within major phyla such as Bacteroidota, Firmicutes, and Fusobacteriota, forming distinguishable enrichment clusters at the genus level. An LDA effect size bar plot further quantified the key discriminatory genera for each group (Figure 7A).

The healthy control group (S1) was primarily enriched in *Fusobacterium*, suggesting a higher representation of anaerobic bacteria associated with the mucosal niche in healthy oral cavities. The benign pulmonary nodule group (S2) was significantly enriched in *Prevotella_7* and *Veillonella*, both commonly found in oral anaerobic ecosystems. This may reflect that the oral microbial structure in individuals with nodules has undergone some degree of alteration, though not yet entering a tumor-associated dysbiotic state. The characteristic genus for the early lung cancer group (S3) was *Streptococcus*. Its significant enrichment suggests a possible phenomenon of dominant bacterial enhancement/colonization expansion in the early stage, consistent with the previously observed decline in α-diversity (Shannon index). This implies that the early-stage oral microbiota may experience a transition characterized by “diversity loss and dominance by specific bacteria.” The advanced lung cancer group (S4) was enriched in *Leptotrichia*, indicating microbial replacement or niche rearrangement associated with advanced disease, suggesting that with increasing tumor burden, the oral microbial structure becomes further complexified and forms new stage-specific characteristics (Figure 7B).

The differential salivary bacteria exhibit a continuous trajectory of change across groups, following the sequence “Control → Benign → Early → Advanced”: transitioning from a healthy structure represented by *Fusobacterium*, to a nodule-type structure characterized by Prevotella_7/Veillonella, to the significant dominance of *Streptococcus* in early-stage lung cancer, and finally forming an advanced-stage characteristic flora represented by *Leptotrichia* in the late stage.

#### 3.4.4 Differential species characteristics of gut microbiota at different disease progression stages (LEfSe analysis)

To further investigate specific bacterial biomarkers across different disease stages, LEfSe analysis was employed to identify bacterial taxa with statistically significant differences among groups from the phylum to species level (LDA score > 3.5, *P* < 0.05). The analysis revealed a significant succession of dominant gut microbial species across the four stages from healthy to advanced lung cancer (Figure 8).

Healthy control group (F1, red): The microbiota enriched in this group primarily consisted of beneficial genera producing short-chain fatty acids. Significantly enriched taxa included *Roseburia*, *Romboutsia*, and the *Ruminococcus torques* group. These bacteria are generally associated with maintaining intestinal barrier function and anti-inflammatory homeostasis. Benign pulmonary nodule group (F2, green): The characteristic markers for this group were relatively singular, with significant enrichment of *Bacteroides plebeius*. This suggests that at the benign lesion stage, the gut microbiota may be in an intermediate state transitioning from health to disease. Early-stage lung cancer group (F3, blue): This group exhibited the most pronounced features of dysbiosis, with cascading enrichment of Proteobacteria and its classes Gammaproteobacteria, orders Enterobacterales, and families Enterobacteriaceae. At the genus level, the opportunistic pathogen *Escherichia-Shigella* showed a very high LDA score approaching 5.0, indicating extremely high abundance. Additionally, *Megamonas* was also significantly increased in this group. This type of alteration, characterized by the expansion of Proteobacteria, typically suggests the formation of an intestinal inflammatory environment. Advanced-stage lung cancer group (F4, orange): As the disease progressed to an advanced stage, the dominant microbiota shifted again. This group was significantly enriched in *Veillonella*, *Gordonibacter* (and its species *Gordonibacter pamelaeae*), Lactobacillaceae, and *Bacteroides coprocola*. Notably, the enrichment of *Veillonella* in advanced-stage samples is directionally consistent with prior lung cancer studies reporting increased abundance of oral-associated taxa, including *Veillonella*, in cancer-related microbial dysbiosis (Souza et al., 2023; Zeng et al., 2023). In particular, experimental work has shown that *Veillonella parvula* can promote lung adenocarcinoma proliferation in model systems (Zeng et al., 2023). Nevertheless, in the context of the present fecal 16S dataset, this finding should be interpreted as an association only; it does not prove oral origin, intestinal colonization dynamics, or a direct tumor-promoting role in our cohort (Li et al., 2024; Souza et al., 2023; Tortora et al., 2023; Yamazaki and Kamada, 2024; Zeng et al., 2023).

#### 3.4.5 Changes in salivary microbiota abundance

To delineate the dynamic changes in the salivary microbial community during disease progression, we analyzed the relative abundance trends of specific bacterial taxa across the four stages: healthy control (S1), benign nodule (S2), early-stage (S3), and advanced-stage lung cancer (S4). The results revealed significant taxon-specific succession in the salivary microbiota with increasing malignancy.

**FIGURE 9:**
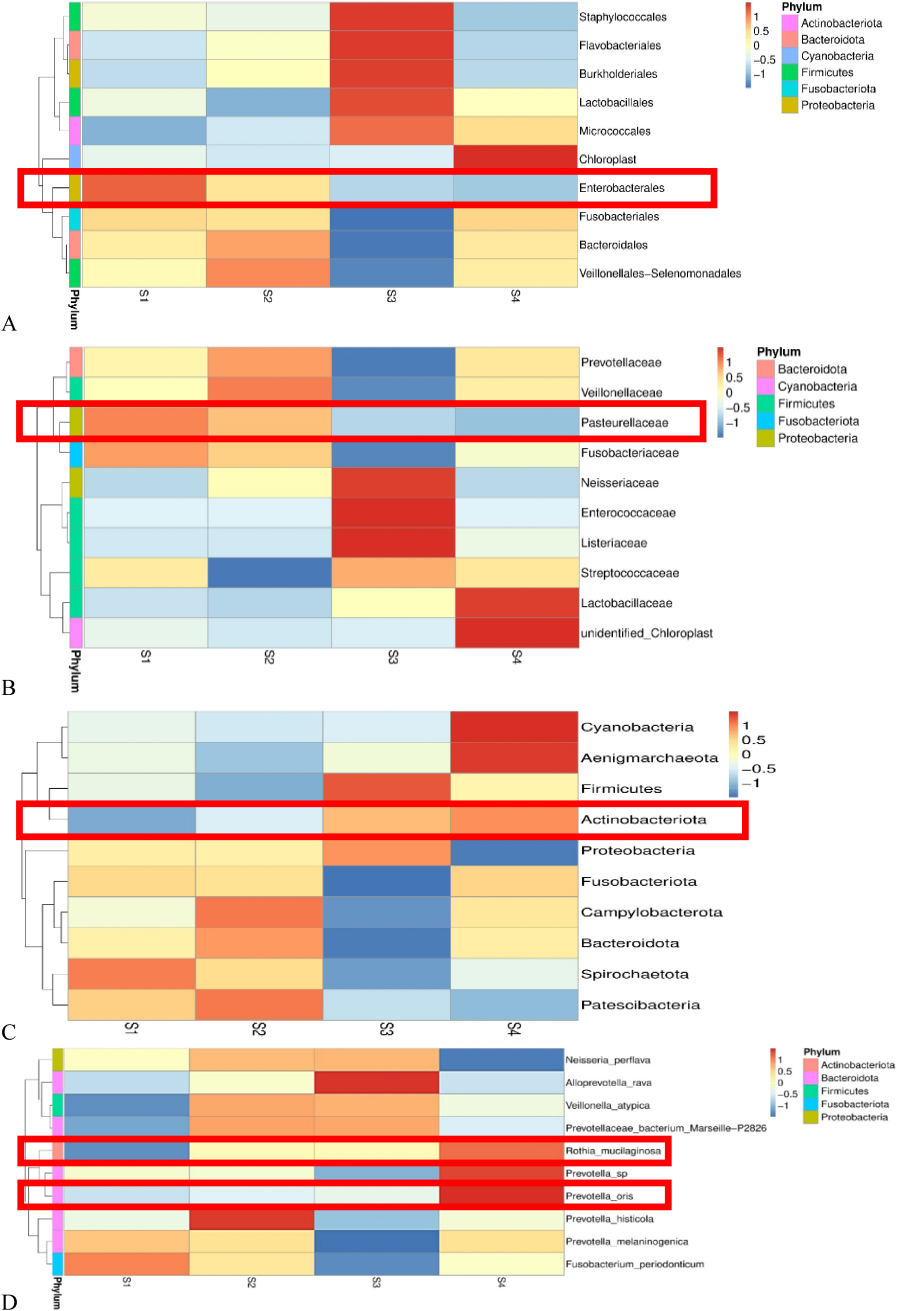
Abundance trends of key salivary bacterial taxa across different disease progression stages. **(A,B)** Bacterial taxa showing a decreasing trend in abundance with disease progression. Boxplots (or line plots) illustrate the changes in relative abundance of Enterobacterales **(A)** and Pasteurellaceae **(B)** across groups. **(C,D)** Bacterial taxa showing an increasing trend in abundance with disease progression. Illustrates the progressive enrichment of Actinobacteriota **(C)**, *Rothia mucilaginosa* **(D)**, and *Prevotella oris* **(D)**.

(1) Depleted Taxa: Certain taxa commonly found in healthy oral cavities showed a decreasing trend in abundance with disease progression. Specifically, the relative abundance of Enterobacterales and Pasteurellaceae was highest in the healthy control group (S1) and gradually decreased with the appearance and worsening of pulmonary lesions, reaching its lowest point in the advanced lung cancer group (S4). This gradient decline suggests that these bacterial groups may be sensitive to changes in the tumor microenvironment or host immune status, with their colonization capacity being suppressed during disease progression.

(2) Enriched Taxa: Conversely, some opportunistic pathogens or anaerobes exhibited characteristics of gradual accumulation with disease progression. At the phylum level, the abundance of Actinobacteriota progressively increased with disease severity. Deeper analysis at the species level revealed a significant linear increasing trend in the abundance of *Rothia mucilaginosa* (belonging to Actinobacteriota) and *Prevotella oris*. The overgrowth of *Rothia mucilaginosa*, a common oral bacterium, in the cancer group may be associated with oral dysbiosis and weakened host immune defense. The gradient increase in *Prevotella oris*, a Gram-negative anaerobe, might indicate a shift in the oral environment toward an inflammatory state more conducive to anaerobic bacterial survival. Trends in the abundance of key salivary bacterial taxa across various stages of disease progression is shown in Figure 9.

Group labels: S1: healthy control; S2: benign pulmonary nodule; S3: early-stage lung cancer; S4: advanced-stage lung cancer.

Statistical analysis: Trend analysis was performed using the Jonckheere-Terpstra test or linear regression models; *P* < 0.05 indicates a statistically significant trend.

#### 3.4.6 Changes in fecal microbiota abundance

**FIGURE 10:**
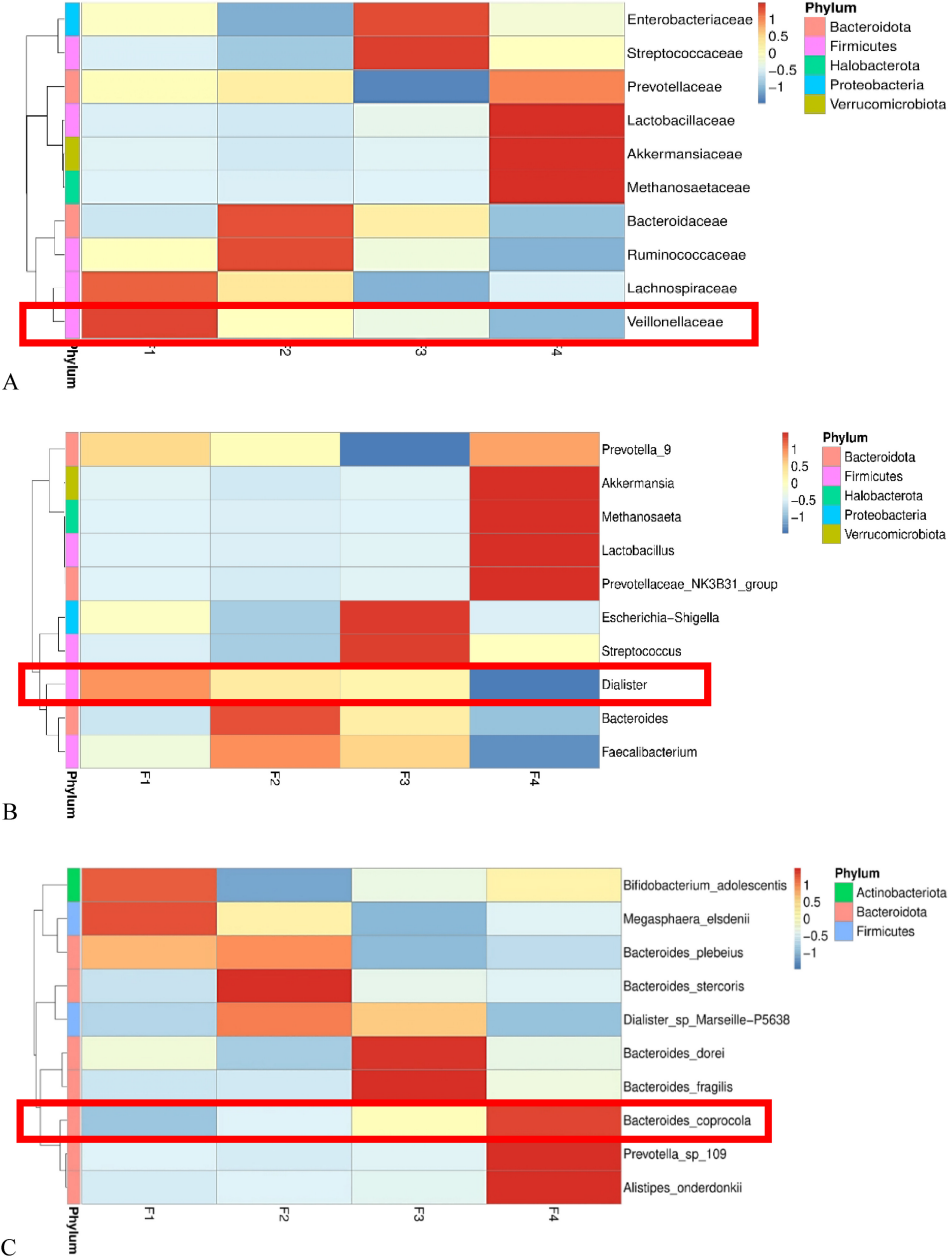
Heatmap showing the abundance evolution of key gut bacterial taxa during disease progression. **(A)** Abundance distribution of *Veillonellaceae* across groups, showing a gradual decrease with disease progression. **(B)** Abundance changes of *Dialister*, showing significant depletion in the advanced lung cancer group. **(C)** Abundance changes of *Bacteroides coprocola*, showing a progressive enrichment trend with disease progression. Each column in the heatmap represents a group (F1: healthy control; F2: benign pulmonary nodule; F3: early-stage lung cancer; F4: advanced-stage lung cancer). Each row represents a bacterial taxon. The color scale represents Z-score normalized relative abundance: red represents high abundance, blue represents low abundance. The color key on the right shows the correspondence between values and colors (range 1 to - 1).

To further explore the dynamic association between gut microbiota and lung cancer stage, we visualized the relative abundance trends of key differential species across the four subgroups using heatmaps. The results showed that specific bacterial taxa exhibited unidirectional successional changes with disease progression (Figure 10).

(1) Depletion Trend of “Health-Related” Bacteria: Analysis revealed that some commensal bacteria with high abundance in healthy controls exhibited a gradient decline in abundance with the appearance and malignant progression of pulmonary nodules. Veillonellaceae: The heatmap showed the highest abundance of this family in the healthy control group (F1, depicted as dark red), transitioning to cool colors (blue) in the early-stage (F3) and advanced-stage (F4) lung cancer groups, suggesting significant inhibition or niche replacement under cancerous conditions (Figure 10A). Dialister: This genus displayed a similar pattern of decline. Although it maintained relatively high levels in the benign nodule group (F2), its abundance sharply decreased to the lowest point (dark blue region) as the disease progressed to advanced lung cancer (F4), indicating that the depletion of this genus may be closely related to changes in the gut microenvironment in advanced cancer (Figure 10B).

(2) Enrichment Trend of “Disease-Related” Bacteria: In contrast to the above trends, certain potential opportunistic pathogens exhibited gradual accumulation with increasing disease severity. Bacteroides coprocola: This species demonstrated the most typical positive succession pattern. In the healthy (F1) and benign nodule (F2) groups, its relative abundance was low (blue background). Upon entering the early-stage lung cancer (F3) phase, abundance began to increase (yellow transition). By the advanced-stage lung cancer (F4) phase, this bacterium showed explosive growth (dark red high-abundance zone). This linear enrichment trend from low to high suggests that *Bacteroides coprocola* may be an important characteristic marker of gut dysbiosis in advanced lung cancer (Figure 10C).

### 3.5 Cross-stage microecological succession trend analysis

To investigate the stage-specific evolution characteristics of the microecosystem during lung cancer progression, participants were divided into four subgroups: healthy control (C), benign nodule (B), early-stage lung cancer (EL), and advanced-stage lung cancer (AL) for trend analysis (Spearman rank correlation and trend statistics).

**FIGURE 11:**
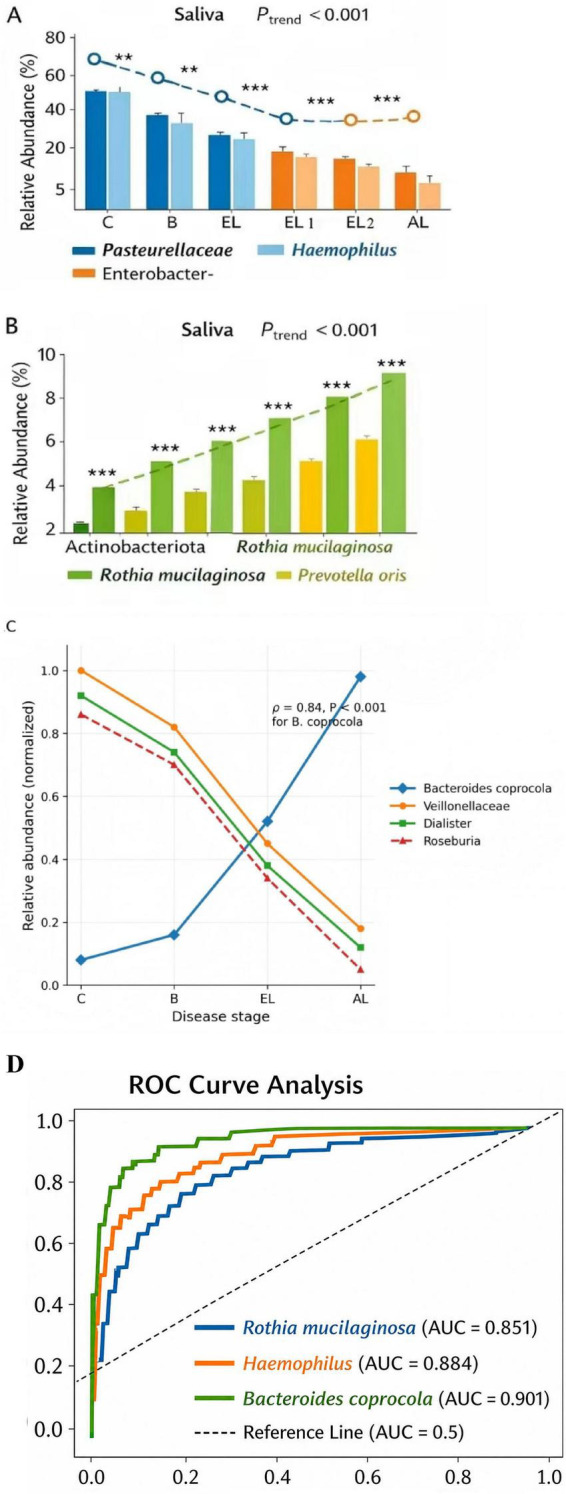
Stage-specific evolution of salivary and gut microbiota during lung cancer progression and diagnostic performance of candidate biomarkers. **(A)** Gradient decline in salivary microbiota: The relative abundances of Pasteurellaceae (primarily the genus *Haemophilus*) and Enterobacterales showed a significant decreasing trend along the disease continuum from healthy controls **(C)** to benign nodule carriers **(B)** and further to lung cancer patients (EL, AL). **(B)** Gradient increase in salivary microbiota: The abundances of Actinobacteriota, *Rothia mucilaginosa*, and *Prevotella oris* increased significantly with advancing disease severity, suggesting that the expansion of these opportunistic pathogens may reflect a shift in the oral environment toward a pro-inflammatory state that parallels lung carcinogenesis. *P* trend < 0.001. **(C)** Succession of gut microbiota: Health-associated taxa, including Veillonellaceae, *Dialister*, and *Roseburia*, progressively decreased across the disease spectrum. In contrast, *Bacteroides* coprocola exhibited a marked gradient increase, with its abundance strongly correlated with lung cancer stage (rho = 0.84, *P* < 0.001), potentially contributing to metabolic remodeling and systemic inflammatory signaling (e.g., via the TLR4 pathway) that facilitates tumor progression. **(D)** ROC curve analysis: Evaluation of the performance of candidate biomarkers in distinguishing non-tumor individuals (C+B) from lung cancer patients (EL+AL). Gut *B. coprocola* achieved the highest diagnostic efficacy as a single marker (AUC = 0.901), followed by salivary *Haemophilus* (AUC = 0.884) and *Rothia mucilaginosa* (AUC = 0.851). Trend analyses were performed using the Jonckheere-Terpstra test or linear regression models. * *P* < 0.05, ** *P* < 0.01, *** *P* < 0.001. Multivariable models confirmed these stage-specific trends remained significant after adjusting for age, sex, and smoking status, reinforcing their potential as robust non-invasive biomarkers.

In saliva samples, the relative abundance of Pasteurellaceae (primarily comprising *Haemophilus*) and Enterobacterales showed a significant gradient decrease (*P*_trend_ < 0.001). Conversely, the abundance of Actinobacteriota, *Rothia mucilaginosa*, and *Prevotella oris* significantly increased with disease progression (C → B → EL → AL) (Figures 11A,B). Notably, the benign nodule group (B) often exhibited intermediate abundance levels for these genera, situated between the C and EL groups.

Loss of Gut Barrier-Associated Microbiota: In fecal samples, the abundance of Veillonellaceae and *Dialister* continuously decreased with disease progression. Particularly, the butyrate-producing beneficial bacterium *Roseburia* was nearly depleted in advanced lung cancer patients (AL). *Bacteroides coprocola* exhibited a very strong gradient increase (Figure 11C), with its relative abundance positively correlated with lung cancer stage (rho = 0.84, *P* < 0.001). This suggests that this bacterium might be involved in metabolic remodeling that promotes tumor progression. To evaluate the potential clinical utility of these differential bacteria, receiver operating characteristic (ROC) curve analysis was performed to assess the efficacy of candidate markers in distinguishing non-tumor individuals (C+B) from lung cancer patients (EL+AL). *Rothia mucilaginosa* and *Haemophilus* in saliva both demonstrated excellent discriminatory ability, with AUC values of 0.851 and 0.884, respectively. *Bacteroides coprocola* in the gut exhibited the highest diagnostic efficacy as a single marker, achieving an AUC of 0.901 (Figure 11D), suggesting its high sensitivity and specificity for non-invasive lung cancer screening. These markers maintained stable discriminatory performance when specifically comparing the benign nodule (B) group with early-stage lung cancer (EL). Furthermore, multivariable regression models confirmed that the stage-specific gradient evolution of R. mucilaginosa and B. coprocola remained statistically significant after adjusting for age, sex, smoking status, and pathological type (adenocarcinoma vs. non-adenocarcinoma). Specifically, the positive association between disease stage and salivary R. mucilaginosa remained significant after adjustment (adjusted *P* = 0.018), and the positive association between disease stage and fecal B. coprocola also remained significant (adjusted *P* = 0.006). Consistently, histology-restricted sensitivity analysis within the adenocarcinoma subset showed the same directional trends for these taxa, supporting that their fluctuations primarily reflect increasing systemic tumor burden rather than differences in histological composition alone.

## 4 Discussion

### 4.1 Lung cancer-associated “oral-gut-lung axis” microbial imbalance: from community structure to systemic immune impact

In this study, analyzing multi-site samples from 111 participants, we observed significant remodeling of both salivary and fecal microbial communities in association with lung cancer. β-diversity analysis based on Unweighted UniFrac distances revealed a clear separation in community structure between the lung cancer group and the non-tumor group, suggesting that lung cancer is not merely a localized neoplastic event but is accompanied by a disruption of systemic microbial homeostasis. This phenomenon may be interpreted within the broader framework of the gut-lung axis and the common mucosal immune system. Recent reviews suggest that oral, gut, and respiratory microbial communities are linked through bidirectional interactions involving microbial translocation, circulating metabolites, and immune crosstalk; however, the magnitude and disease-specific relevance of these interactions remain context dependent (Li et al., 2024; Souza et al., 2023; Tortora et al., 2023; Yamazaki and Kamada, 2024). In this context, microorganisms and their metabolites (e.g., SCFAs) may influence distal pulmonary immune tone through circulation and immune-cell trafficking, thereby potentially shaping inflammatory status and the tumor microenvironment, although such mechanisms were not directly examined in the present study (Budden et al., 2017; Li et al., 2024; Souza et al., 2023). Consistent with prior research, patients with lung cancer harbor identifiable gut microbial signatures with potential for early prediction. For instance, Haberman et al. (2023) analyzed the gut microbiota of patients with early-stage NSCLC and identified microbial signatures capable of distinguishing them from healthy controls, demonstrating reasonable discriminatory accuracy in a validation cohort and providing population-level evidence for the “lung cancer-gut microbiota” association.

### 4.2 Salivary microbiota: dynamic fluctuations of “decline in early stage—recovery in advanced stage” and underlying mechanisms

Stratification by disease stage revealed phase-specific fluctuations in the salivary microecology. The overall structure remained relatively stable in the benign nodule stage. However, the early-stage lung cancer group exhibited a decline in diversity accompanied by enrichment/replacement of specific dominant bacteria, while the advanced-stage group displayed higher heterogeneity and a more “complex” community structure (more dispersed PCoA distribution). This “decline followed by increase” pattern should be interpreted cautiously. In the early stage, the enrichment of *Streptococcus* may reflect a shift in oral ecological balance under altered mucosal or systemic conditions, rather than a directly demonstrated suppressive effect on other commensals. Likewise, the enrichment of *Leptotrichia* in advanced disease may indicate ecological replacement under a more perturbed host environment, but our cross-sectional data do not allow causal inference regarding barrier disruption, competitive exclusion, or immune escape. Therefore, these stage-specific salivary changes are best regarded as descriptive microbial correlates of disease progression, with mechanistic interpretation remaining speculative.

Trend analysis provided clear evidence of directional succession. Pasteurellaceae (primarily *Haemophilus*) and Enterobacterales decreased significantly along the C→ B→EL→AL continuum, whereas Actinobacteriota, *Rothia mucilaginosa*, and *Prevotella oris* increased significantly with disease progression. These findings resonate with existing studies on salivary/oral microbiota in lung cancer. For example, Yang et al. (2018) observed salivary dysbiosis in a cohort of non-smoking women with lung cancer, correlating with immune markers, supporting the notion that the salivary microecology can reflect tumor-associated immune status.

### 4.3 Gut microbiota: defensive barrier decline and gradient enrichment of “disease-associated” bacteria

The gut microbiota exhibited an even more “directional” succession pattern. Veillonellaceae and *Dialister* consistently decreased with disease progression, with butyrate-producing commensals such as *Roseburia* being nearly depleted in the advanced stage, suggesting a decline in gut barrier function and immune homeostasis. At the mechanistic level, SCFAs, particularly butyrate, are crucial metabolites for maintaining intestinal mucosal integrity and regulating systemic immunity. Relevant reviews have highlighted that SCFAs can influence pulmonary immune responses and inflammatory susceptibility, serving as key chemical mediators of the “gut-lung axis” (Liu et al., 2021). Of greater clinical significance, *Bacteroides coprocola* exhibited a pronounced stage-dependent increase, positively correlated with disease stage. Within the broader microbiota-tumor framework, previous studies have shown that microbial products and host pattern-recognition pathways can contribute to inflammatory remodeling in cancer-related settings (Arthur et al., 2012; Souza et al., 2023; Zeng et al., 2023). Arthur et al. (2012) provided experimental evidence that intestinal inflammation can reshape microbial composition and promote tumorigenesis. In the present study, the gradient enrichment of *Bacteroides coprocola* is consistent with the possibility that disease progression is accompanied by microbiota-associated inflammatory or metabolic remodeling; however, our data do not demonstrate that this species directly activates TLR4 signaling, nor do they establish a causal microbiota-inflammation-tumor loop in lung cancer. Accordingly, the potential involvement of TLR4-related or other innate immune pathways should be regarded as a hypothesis informed by prior literature rather than a direct conclusion of this study (Souza et al., 2023; Zeng et al., 2023). Therefore, the observed combination of “commensal depletion” and “disease-associated bacterial enrichment” likely represents a microecological phenotype of systemic inflammation and metabolic alterations during lung cancer progression.

### 4.4 Incorporating benign nodule controls: disentangling “tumor-specific alterations” from “nodule/inflammation-associated changes”

In non-invasive lung cancer screening research, the traditional “lung cancer vs. healthy control” paradigm often inadequately replicates the real-world clinical challenge of nodule differentiation. This approach risks conflating “benign nodule/inflammation-related changes” with tumor-specific alterations, potentially leading to diminished biomarker performance when extrapolated to clinical settings (British Thoracic Society, 2015; Choi et al., 2018). By introducing benign nodules as a non-tumor control, this study established an evaluation system more closely aligned with the authentic clinical scenario of “differentiating benign from malignant nodules,” thereby methodologically aiding in the disambiguation of “nodule/inflammation-associated changes” from true tumor-specific features (British Thoracic Society, 2015; Choi et al., 2018). A further concern in interpreting the early-versus-advanced comparisons is the unequal histological composition of these two subgroups, particularly the higher proportion of squamous cell carcinoma and other non-adenocarcinoma histologies in the advanced-stage group. Because different histological subtypes may themselves be associated with distinct host inflammatory states and mucosal microbial ecosystems, the observed separation in Figure 6 could theoretically reflect histology rather than stage. To address this issue, we explicitly incorporated pathological type into multivariable models and additionally performed sensitivity analyses restricted to adenocarcinoma, the dominant subtype in both groups. Importantly, the overall stage-dependent microbial separation and the directional changes of key taxa remained detectable after these analyses. These findings support the interpretation that tumor progression is a major driver of the observed microbial remodeling, although a modest contribution from subtype-specific biology cannot be fully excluded.

Our findings revealed that while the benign nodule group’s overall β-diversity spatial distribution was generally closer to that of healthy controls, notable shifts in the abundance of specific key genera were already evident. Importantly, these genera exhibited significant gradient evolution across the C→B→EL→AL disease spectrum: *Pasteurellaceae* (e.g., *Haemophilus*) in saliva showed a gradient decrease with disease progression, while *Rothia mucilaginosa* increased significantly; in fecal samples, *Bacteroides coprocola* demonstrated a strong gradient increase. This “intermediate state” phenomenon suggests that remodeling of the microecosystem may occur synchronously with, or even precede, the imaging-detectable emergence of lesions and their invasive transformation. Notably, although lung cancer is histologically heterogeneous, emerging evidence indicates that the core microbial dysbiosis associated with malignancy is relatively conserved across different pathological subtypes, such as adenocarcinoma and squamous cell carcinoma. This suggests that the systemic “cancer signal” and the physiological stress associated with increasing tumor burden act as more dominant drivers of oral-gut remodeling than subtype-specific histological features. Previous oral and gut microbiome studies have also suggested that microbial characteristics can exhibit recognizable differences at the early stages of lung carcinogenesis, lending theoretical feasibility to their use for “risk stratification/early warning” (Leng et al., 2021; Vogtmann et al., 2022).

ROC analysis revealed that the candidate microbial markers possessed excellent discriminatory power in distinguishing non-tumor individuals from lung cancer patients. Salivary markers *R. mucilaginosa* and *Haemophilus* achieved AUCs of 0.851 and 0.884, respectively; the gut marker *B. coprocola* attained an AUC of 0.901. More importantly, these markers maintained stable discriminatory performance specifically when differentiating benign nodules from early-stage lung cancer—a notoriously difficult clinical dilemma. This suggests their high sensitivity to tumor-related biological signals. Related research also emphasizes that including “benign lung disease/benign nodules” as controls helps enhance model suitability for real-world clinical tasks (Chen et al., 2023; Lee et al., 2016).

Despite the impressive AUC values indicating substantial clinical potential, the path toward translation warrants cautious consideration. Future research should prioritize three key directions: (1) Multicenter validation: Assessing marker robustness and reproducibility in prospective cohorts across diverse geographical regions, ethnicities, and dietary patterns. (2) Multimodal integration: Evaluating the incremental value of microbial markers alongside low-dose computed tomography (LDCT) and clinical risk factor models (e.g., Brock/PanCan nodule malignancy risk models) and exploring their potential as adjunctive decision-making tools (Cui et al., 2019; de Koning et al., 2020; National Lung Screening Trial Research Team et al., 2011). (3) Mechanistic elucidation: Further clarifying the molecular mechanisms by which key bacteria such as *B. coprocola* may promote stage-specific lesion progression via immune-metabolic axes (e.g., chronic inflammatory signaling triggered by bacterial components or metabolite changes). Reviews on the “gut-lung axis” also indicate that microbial metabolites and immune regulation likely jointly shape the pulmonary inflammatory/tumor microenvironment (Dang and Marsland, 2019; Perl et al., 2025).

Several limitations of this study should be acknowledged. First, the retrospective and cross-sectional nature of this study inherently precludes any conclusions regarding causality; consequently, it remains unclear whether the observed oral-gut dysbiosis acts as a functional driver of lung tumorigenesis or represents a secondary epiphenomenon resulting from the systemic physiological and immune shifts associated with cancer progression. Second, the single-center nature and modest sample size (*N* = 111) limit the generalizability of our findings. The cohort was recruited from a single institution in Beijing, which introduces potential population-specific biases related to local environmental exposures and the Northern Chinese dietary pattern. Consequently, these microbial signatures may not fully represent populations with different ethnic backgrounds or lifestyles, and external validation in multi-ethnic, geographically diverse prospective cohorts is essential to confirm the universal applicability of these biomarkers. While our *post-hoc* power analysis confirmed that the large effect sizes for key genera (*f*^2^ > 0.35) provided sufficient power (>80%) to detect differences despite the small benign group (*n* = 18), the inherent imbalance in group sizes could still influence the stability of our LEfSe and ROC models. To mitigate this, we employed cross-validation techniques and relied on rank-based statistics to ensure that the identified signatures were not driven by outliers in the smaller subgroups. Consequently, the identified microbial signatures and their high diagnostic performance require rigorous external validation in large-scale, geographically distinct cohorts to ensure their stability across different demographic and clinical backgrounds. Third, the cross-sectional design, characterized by single time-point sampling, lacks the longitudinal follow-up necessary to establish a clear temporal sequence of microbial shifts. This limitation hinders our capacity to determine if specific microbial alterations precede tumor initiation, which is essential for definitively identifying early-warning biomarkers rather than mere indicators of established disease. Fourth, the use of 16S rRNA sequencing provides limited taxonomic resolution and lacks direct functional data, which prevents us from definitively characterizing the metabolic pathways involved in tumor promotion. Future research should employ shotgun metagenomics and metabolomics to achieve strain-level identification and to clarify the functional roles of microbial metabolites (e.g., specific SCFAs or secondary bile acids) within the gut-lung axis. Finally, while we applied multivariable models to adjust for smoking status, residual confounding from unmeasured lifestyle factors cannot be entirely ruled out. Specifically, detailed data on smoking intensity (e.g., pack-years), duration of exposure, and granular dietary intake (such as fiber and protein proportions)—all of which are known to modulate the oral-gut axis—were not comprehensively analyzed. Future longitudinal studies should incorporate standardized dietary logs and detailed smoking history to more accurately isolate the independent effect of lung carcinogenesis on the microbiome. These unmeasured lifestyle factors might influence the oral-gut microecology and should be more comprehensively documented in future longitudinal studies. Although the observed gradient changes across the disease spectrum suggest tumor-specific contributions to microecological remodeling beyond smoking and histological variations alone, the relatively limited sample size within individual histological subtypes still constrains the depth of subtype-stratified analysis. Therefore, validation in larger, histology-matched, multicenter cohorts will be necessary to determine whether the identified microbial signatures are universally stage-dependent across pathological subtypes or partially subtype-specific. However, given that existing literature often reports overlapping microbial signatures between lung adenocarcinoma and squamous cell carcinoma, our findings reinforce the notion that disease progression is the primary force shaping the systemic microecology.

In addition, because this study used a cross-sectional 16S rRNA sequencing design, it cannot determine whether specific taxa are causal drivers, passengers, or merely correlates of tumor progression, nor can it directly resolve oral-to-gut translocation or pathway-level mechanisms. These questions will require longitudinal sampling, metagenomic validation, and experimental studies.

## 5 Conclusion

This study establishes that lung cancer is associated with significant shifts in the β-diversity of both oral and gut microbiota, indicating the occurrence of systemic microbial remodeling. Trend analysis further revealed gradient evolution along the C→B→EL→AL continuum: *Pasteurellaceae* (primarily *Haemophilus*) in saliva significantly decreased, while *Rothia mucilaginosa* and *Prevotella oris* significantly increased; in the gut, *Veillonellaceae* progressively declined, whereas *Bacteroides coprocola* exhibited a strong gradient increase positively correlated with disease stage. The “intermediate state” characteristics observed in the benign nodule group provide a biological basis for risk warning. Candidate markers demonstrated excellent diagnostic efficacy in distinguishing non-tumor individuals (C+B) from lung cancer patients (EL+AL) (salivary *R. mucilaginosa* AUC 0.851, gut *B. coprocola* AUC 0.901) and maintained stable performance in the challenging task of differentiating benign nodules from early-stage lung cancer. In summary, lung cancer progression is accompanied by stage-specific remodeling of the “oral-gut” microecology. Incorporating benign controls facilitates the extraction of more clinically relevant tumor signals. Future efforts should focus on conducting external validation of these markers in large, multi-ethnic, and multicenter prospective cohorts to mitigate potential population bias, while integrating multi-omics technologies with multimodal clinical models to advance their translation into robust tools for non-invasive screening and risk stratification.

## Data Availability

The raw data supporting the conclusions of this article will be made available by the authors, without undue reservation.
