# Supplementary material for: A comparative study of oral and gut microbiota for differentiating benign pulmonary nodules from lung cancer
Source: Front Microbiol. 2026 May 19;17:1824419. doi: 10.3389/fmicb.2026.1824419 (PMC13226548; doi:10.3389/fmicb.2026.1824419)
Supplement: Supplementary file 1 [file Supplementary_file_1.docx]

**Supplementary Table 1. Covariate-adjusted differential abundance results identified by MaAsLin2.**

| **Taxon** | **Sample type** | **Comparison** | **Direction** | **Coefficient** | **Adjusted P value** | **Covariates included** |
| --- | --- | --- | --- | --- | --- | --- |
| Haemophilus | Saliva | Lung cancer vs non-tumor | Decreased in lung cancer | -0.84 | 0.021 | age, sex, smoking status, pathological type |
| Rothia mucilaginosa | Saliva | Advanced-stage trend | Increased with stage | 0.73 | 0.018 | age, sex, smoking status, pathological type |
| Bacteroides coprocola | Feces | Lung cancer vs non-tumor | Increased in lung cancer | 0.91 | 0.006 | age, sex, smoking status, pathological type |
| Bacteroides coprocola | Feces | Advanced-stage trend | Increased with stage | 0.88 | 0.006 | age, sex, smoking status, pathological type |
